# Taxonomic and molecular characterization of a new entomopathogenic nematode species,* Heterorhabditis casmirica* n. sp., and whole genome sequencing of its associated bacterial symbiont

**DOI:** 10.1186/s13071-023-05990-z

**Published:** 2023-10-25

**Authors:** Aashaq Hussain Bhat, Ricardo A. R. Machado, Joaquín Abolafia, Alba N. Ruiz-Cuenca, Tarique Hassan Askary, Fuad Ameen, Wasim Muzamil Dass

**Affiliations:** 1 Department of Biosciences, University Center for Research and Development, Chandigarh University, Gharuan, Mohali, Punjab 140413 India; 2 Experimental Biology Research Group, Institute of Biology, Faculty of Sciences, University of Neuchâtel, Neuchâtel, 2000, Switzerland; 3 Departamento de Biología Animal, Biología Vegetal y Ecología, Universidad de Jaén, Campus ‘Las Lagunillas’, Jaén, 23071, Spain; 4 Division of Entomology, Faculty of Agriculture, Sher-e-Kashmir University of Agricultural Sciences and Technology of Kashmir, Wadura Campus, Sopore, 193201, Jammu and Kashmir India; 5 Department of Botany and Microbiology, College of Science, King Saud University, 11451 Riyadh, Saudi Arabia; 6 Department of Zoology, University of Kashmir, Srinagar, 190006 Jammu and Kashmir India

**Keywords:** Entomopathogenic nematodes, Biological control agents, Species description, Nematode morphology, Phylogenetics, Taxonomy, *Photorhabdus*

## Abstract

**Background:**

Nematodes of the genus *Heterorhabditis* are important biocontrol agents as they form a lethal combination with their symbiotic *Photorhabdus* bacteria against agricultural insect pests. This study describes a new species of *Heterorhabditis*.

**Methods:**

Six *Heterorhabditis* nematode populations were recovered from agricultural soils in Jammu and Kashmir, India. An initial examination using mitochondrial and nuclear genes showed that they belong to a new species. To describe this new species, a variety of analyses were conducted, including reconstructing phylogenetic relationships based on multiple genes, characterizing the nematodes at the morphological and morphometric levels, performing self-crossing and cross-hybridization experiments, and isolating and characterizing their symbiotic bacteria.

**Results:**

The newly discovered species, *Heterorhabditis casmirica* n. sp., shares 94% mitochondrial cytochrome C oxidase subunit I gene (*COI*) sequence identity with *Heterorhabditis bacteriophora* and *Heterorhabditis ruandica*, and 93% with *Heterorhabditis zacatecana*. Morphologically, it differs from *H. bacteriophora* in its infective juvenile phasmids (present vs. inconspicuous) and bacterial pouch visibility in the ventricular portion of the intestine (invisible vs. visible); genital papilla 1 (GP1) position (at manubrium level vs. more anterior), and in its* b* ratio (body length/neck length),* c* ratio (tail length/bulb width), and* D*% [(excretory pore/neck length) × 100]. Other morphological differences include anterior end to the nerve ring distance (77–100 vs. 121–130 μm),* V*% [(anterior end of vulva/body length) × 100] (46–57 vs. 41–47) in hermaphroditic females; rectum size (slightly longer than the anal body diameter vs. about three times longer), phasmids (smaller vs. inconspicuous), body length (0.13–2.0 vs. 0.32–0.39 mm), body diameter (73–150 vs. 160–220 μm), anterior end to the excretory pore distance (135–157 vs. 174–214 μm), and demanian ratios in amphimictic females. Morphological differences with *H. ruandica* and *H. zacatecana* were also observed. Furthermore, *H. casmirica* n. sp. did not mate or produce fertile progeny with other *Heterorhabditis* nematodes reported from India. It was also discovered that *H. casmirica* n. sp. is associated with *'Photorhabdus laumondii * subsp. *clarkei* symbiotic bacteria.

**Conclusions:**

The discovery of *H. casmirica* n. sp. provides novel insights into the diversity and evolution of *Heterorhabditis* nematodes and their symbiotic bacteria. This new species adds to the catalog of entomopathogenic nematodes in India.

**Graphical Abstract:**

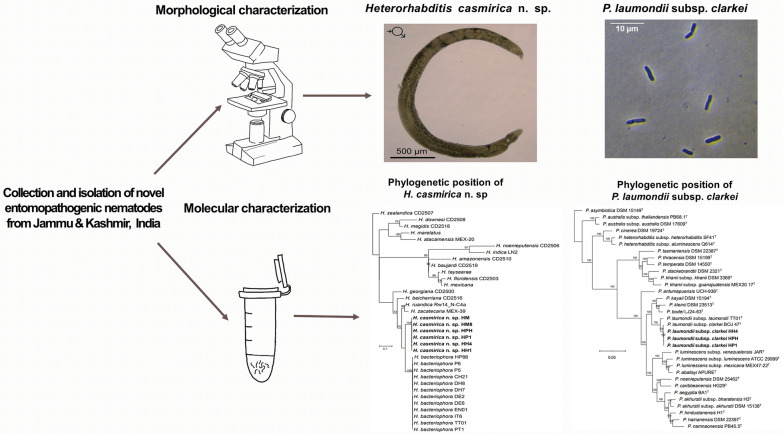

**Supplementary Information:**

The online version contains supplementary material available at 10.1186/s13071-023-05990-z.

## Background

Entomopathogenic nematodes belonging to the families Heterorhabditidae and Steinernematididae are highly effective biocontrol agents against agricultural pests. These nematodes have independently evolved mutual associations with insect pathogenic bacteria of the genera *Photorhabdus* and *Xenorhabdus*, respectively [1–4]. At the infective juvenile (IJ) stage, these nematodes, which reside in the soil, actively search for insect hosts [5]. When an appropriate host is located, the IJs penetrate the insect body through natural openings or by directly breaking through the cuticle. They then release their bacterial symbionts upon sensing unknown chemical cues in the hemolymph [6, 7]. The bacteria multiply and produce virulence factors and toxins that kill the infected host [8–10]. Furthermore, the bacteria secrete exoenzymes that degrade the insect tissues and produce several metabolites essential for nematode growth, development, and reproduction [11, 12]. The bacteria also produce potent secondary metabolites that act as antibiotics and deter scavenging arthropods. Upon resource depletion, the new generation of nematodes disperses in search of new hosts [9, 13].

*Heterorhabditis* species are generally more virulent than those of *Steinernema* [14]. However, they are less speciose than *Steinernema* [15, 16]. Despite this, new valid species of *Heterorhabditis* are often described and added to the list. The genus *Heterorhabditis* comprises 21 valid species, including two recently described species, *Heterorhabditis ruandica* from Rwanda and *Heterorhabditis zacatecana* from Mexico [15, 17]. Most of the valid species described so far have been molecularly characterized, except for *Heterorhabditis egyptii* [18] and *Heterorhabditis hambletoni* [19], which have only been morphologically characterized. The genus *Heterorhabditis* is globally distributed, although some species are only reported in certain geographic regions. In India, for instance, three species of *Heterorhabditis* have been documented so far: *Heterorhabditis indica* [20, 21], *Heterorhabditis bacteriophora* [22], and *Heterorhabditis baujardi* [23]. *Heterorhabditis indica*, described by Poinar et al. [20], is the only new species of the genus *Heterorhabditis* reported from India to date.

In this study, we present the discovery of, and characterize, a new entomopathogenic nematode species, *Heterorhabditis casmirica* n. sp., and its symbiotic bacteria, recovered from the union territory of Jammu and Kashmir, India. Our study contributes to the characterization of soil biodiversity in general and advances our efforts to understand the biodiversity of an important group of biological control agents, which are essential tools for eco-friendly and sustainable agricultural practices.

## Methods

### Nematode origin

Six populations of nematodes, namely HM, HM8, HP1, HPH, HH1, and HH4, were obtained from soil samples collected in the northwestern part of the union territory of Jammu and Kashmir, India. The samples were collected from soils around the roots of walnut and willow trees in the Anantnag district (Global Positioning System coordinates 33.828914, 75.100091; altitude 1606 m above sea level). Each one of these six populations was isolated from different soil samples. Each soil sample was separated by about 2 km from each other. Nematodes were isolated from soil samples using *Corcyra cephalonica* as a bait insect. Insects with nematode infestation symptoms were washed with double distilled H_2_O, sterilized with 0.1% NaOCl_2_, and then placed in White traps to recover the new generation of IJs [24]. Recovered nematodes were reared using *Galleria mellonella* larvae as hosts under laboratory conditions [25, 26]. The IJs were stored in 250-mL tissue culture flasks in a biological oxygen demand incubator at 15 °C [27, 28]. The new species has been registered at ZooBank under urn:lsid:zoobank.org:pub:BBFC7CC6-7294-4548-AA7F-5CD5293E4103.

### Nematode morphological and morphometric characterization, light and scanning electron microscopy

Hermaphroditic females, males and amphimictic females were obtained by dissecting *G. mellonella* cadavers in Ringer’s solution 4 and 6 days after infestation, respectively [26, 28]. The IJs were collected from White traps after emerging from the *G. mellonella* cadavers. The nematodes were then killed with hot water, fixed in TAF solution (2 mL triethanolamine, 7 mL of 40% commercial formaldehyde solution, and 91 mL distilled water), transferred to anhydrous glycerin, and mounted on permanent glass slides with additional layers of paraffin wax to prevent flattening during microscopy [29, 30]. Morphological measurements (in micrometers) were taken using Nikon DS-L2 image acquisition software on a phase-contrast microscope (Nikon Eclipse 80i). Twenty specimens at each developmental stage were measured. Light microscopy (LM) and scanning electron microscopy (SEM) photographs were obtained using various nematological techniques detailed by Abolafia [31]. In brief, nematodes fixed in 4% formalin solution were processed to anhydrous glycerin using Siddiqi’s method with lactophenol-glycerin solutions [32]. Subsequently, the nematodes were permanently mounted on glass microscope slides using the glycerin-paraffin method [33, 34]. The LM photographs were captured using a Nikon Eclipse 80i microscope (Olympus, Tokyo, Japan) with differential interference contrast optics and a Nikon Digital Sight DS-U1 camera. For SEM, nematodes preserved in glycerin were removed from permanent microscope slides by removing the cover glass, rehydrated in distilled water, dehydrated in a graded ethanol–acetone series, critically point dried with liquid CO_2_, mounted on SEM stubs with copper tape, coated with gold in a sputter coater, and finally observed with a Zeiss Merlin microscope (5 kV) (Zeiss, Oberkochen, Germany) [35]. The LM and SEM micrographs, obtained at different magnifications for each structure, were processed and combined using Adobe Photoshop Creative Suite (Microsoft, Redmond, WA).

Comparisons were made between all the valid described species of *Heterorhabditis* based on morphological, morphometric and molecular characters, using the keys published by Machado et al. [17]. Demanian indices and other ratios were calculated following the method outlined by de Man [36]. The stoma morphology was described using the terminology provided by De Ley et al. [37], the spicule and gubernaculum morphology was described using the terminology established by Abolafia and Peña-Santiago [38] and the terminology for pharynx follows the proposals of Bird and Bird [39] and Baldwin and Perry [40].

### Self-crossing and cross-hybridization experiments

Self-crossing and cross-hybridization experiments were carried out on lipid agar plates following the methodology described by Dix et al. [41]. *Heterorhabditis casmirica* n. sp. isolates HM, HM8, HP1, HPH, HH1, and HH4 were crossed with each other and allowed to interact with Indian populations of *H. bacteriophora* (P4, P5 and KAS), *H. indica* (TH7, TH8 and TH9) and *H. baujardi* (HeTD4) nematodes. Control experiments were also conducted by self-crossing all the nematode species/strains. In each experiment, 20 second-generation males and 20 second-generation virgin females of each species were placed on 35-mm-diameter lipid agar plates and incubated at 25 °C. Progeny production was observed daily for 7 consecutive days. The experiments were conducted twice under the same conditions.

### Nematode molecular characterization and phylogenetic relationships

Genomic DNA was extracted from individual hermaphroditic females isolated from insect cadavers infested with *H. casmirica* n. sp. HM, HM8, HP1, HPH, HH1, or HH4, as described [42]. Briefly, individual virgin females were washed separately with Ringer’s solution and then washed in phosphate-buffered saline (pH 7.2). Virgin females were then individually transferred to sterile polymerase chain reaction (PCR) tubes (0.2 mL) containing 20 μL extraction buffer (17.6 μL nuclease-free distilled H_2_O, 2 μL of 5X PCR buffer, 0.2 μL 1% Tween, and 0.2 μL proteinase K). Samples were frozen at −20 °C for 60 min or overnight and then immediately incubated in a PCR thermocycler at 65 °C for 1.2 h, followed by incubation at 95 °C for 10 min. The lysates were cooled on ice and centrifuged at 6500* g* for 3 min. The resulting supernatants were used as DNA templates to amplify different taxonomically relevant gene markers. A fragment of ribosomal rRNA (rRNA) containing the internal transcribed spacer (ITS) regions (ITS1-5.8S-ITS2) was amplified using primers 18S (5′-TTGATTACGTCCCTGCCCTTT-3′) (forward) and 28S (5′-TTTCACTCGCCGTTACTAAGG-3′) (reverse) [43]. A fragment of rRNA containing the D2–D3 regions of the 28S rRNA was amplified using primers D2F (5′-CCTTAG TAACGGCGAGTGAAA-3′) (forward) and 536 (5′-CAGCTATCCTGAGGAAAC-3′) (reverse) [44]. The 12S mitochondrial gene was amplified using primers 505F (5′-GTTCCAGAATAATCGGCTAGAC-3′) (forward) and 506R (5′-TCTACTTTACTACAACTTACT CCCC-3′) (reverse) [44] and the mitochondrially encoded cytochrome oxidase subunit I gene (*MT-COI*) was amplified using primers HCF (5′-TTACATGATACTTATTATG-3′) (forward) and HCF (5′-CTGATAACTGTGACCAAATACATA-3′) (reverse) [45]. The PCR reactions consisted of 2 µL of DNA extract, 12.5 µL of DreamTaq Green PCR Master Mix (Thermo Scientific, USA), 0.75 µL of each forward and reverse primer at 10 µM and 9 µL of nuclease-free distilled H_2_O. The PCR reactions were performed using a thermocycler (Applied Biosystems Veriti 96-Well Thermal Cycler) with the following settings: (i) for ITS, D2–D3 and 12S—one cycle of 3 min at 94 °C followed by 35 cycles of 30 s at 94 °C, 30 s at 50 °C, 1 min 30 s at 72 °C, followed by a single final elongation step at 72 °C for 20 min; (ii) for the *MT-COI* gene—one cycle of 3 min at 94 °C followed by 38 cycles of 10 s at 94 °C, 30 s at 40 °C, 60 s at 72 °C, followed by a single final elongation step at 72 °C for 10 min [46]. PCR was followed by electrophoresis (45 min, 100 V) of 5 μL of PCR products in a 1% Tris–boric acid–ethylenediaminetetraacetic acid-buffered agarose gel stained with SYBR Safe DNA Gel Stain (Invitrogen, Carlsbad, CA). PCR products were purified using the FastGene Gel/PCR extraction kit (Nippon Genetics, Japan) and sequenced using reverse and forward primers by Sanger sequencing (Bioserve, Hyderabad, India). The obtained sequences were manually curated, trimmed and deposited at the National Center for Biotechnology Information (NCBI) under the accession numbers given in Additional file 1: Table S4. To complete this data set and to obtain genomic sequences of nematodes that belong to all the valid described species of the genus *Heterorhabditis*, we searched the database of the NCBI by using the Basic Local Alignment Search Tool and the accession numbers of the sequences obtained previously [17, 47]. The resulting sequences were used to reconstruct phylogenetic relationships by the maximum likelihood method based on the following nucleotide substitution models: Tamura–Nei (TN93+G+I) (*MT-COI*) and Kimura 2-parameter (K2+G) (D2–D3) (ITS). To select the best substitution models, best-fit nucleotide substitution model analyses were carried out in MEGA 11 [48–51]. Sequences were aligned with MUSCLE (v3.8.31) [52]. The trees with the highest log likelihood are shown. The percentage of trees in which the associated taxa clustered is shown next to the branches. Initial tree(s) for the heuristic search were obtained automatically by applying neighbor–joining and BIONJ algorithms to a matrix of pairwise distances estimated using the maximum composite likelihood approach, and selecting the topology with a superior log-likelihood value. In some cases, a discrete gamma distribution (+G) was used to model evolutionary rate differences between sites, and the rate variation model allowed for some sites to be evolutionarily invariable (+I). The trees are drawn to scale, with branch lengths measured in the number of substitutions per site. Graphical representation and edition of the phylogenetic trees were performed with Interactive Tree of Life v3.5.1 [53, 54].

### Symbiotic relationships

The *Photorhabdus* entomopathogenic bacteria associated with the different *H. casmirica* n. sp. nematode populations were isolated as described previously [55, 56]. Briefly, larvae of *G. mellonella* (Lepidoptera: Pyralidae) were exposed to 100 nematode IJs. Three to 4 days later, insect cadavers were surface sterilized and cut open with a surgical blade. Bacteria-digested internal organs were spread onto Luria—Bertani (LB) agar plates and incubated at 28 °C for 24–48 h. *Photorhabdus*—like colonies were then streaked on fresh LB agar plates until monocultures were obtained. A single primary form colony was then selected and used for further experiments. Bacteria primary forms were determined by examining colony morphology, colony texture, pigment production, and bioluminescence. The strains were further subcultured and maintained on LB agar plates at 28 °C. An initial molecular characterization, using 16S rRNA gene sequences, was carried out to determine the taxonomic affiliation of the obtained bacterial cultures as described previously [3, 4, 17, 56]. Phylogenetic reconstruction and sequence comparisons based on whole genome sequences were carried out to confirm the taxonomic affiliation of the obtained bacterial cultures as described previously [3, 55, 56]. Briefly, genomic DNA was extracted and purified using the GenElute Bacterial Genomic DNA Kit (Sigma-Aldrich, Switzerland) following the manufacturer’s instructions. The resulting DNA was used for library preparation using the TruSeq DNA PCR-Free LT Library Prep (FC-121-3003) kit. Indexed libraries were then pooled at equimolar concentrations and sequenced [2 × 150 base pairs (bp)] on an Illumina HiSeq 3000. Raw Illumina reads were quality trimmed using Trimmomatic 0.39 [57]. The resulting reads were assembled with SPAdes 3.14.1 (*k*-mer sizes of 31, 51, 71, 91, and 111 bp) [58]. Scaffolds with a mean read depth smaller than 20% of the median read depth of the longer scaffolds (≥ 5000 bp) as well as scaffolds that were shorter than 200 bp were removed. The final assemblies were polished using Pilon 1.22 [59]. Phylogenetic relationships were reconstructed based on the assembled genomes and the genome sequences of all valid published species of the genus [3, 55, 56]. For this, core genome alignments were created using Roary 3.6.2 [60]. Based on this alignment, a maximum likelihood tree was constructed using Fasttree 2.1.10 based on the Jukes–Cantor plus CAT nucleotide evolution model [61].

## Results and discussion

Six populations of *Heterorhabditis* nematodes (HM, HM8, HP1, HPH, HH1, and HH4) were isolated from agricultural soils in Kashmir, India. Initial molecular and morphological characterization showed that they are genetically identical, morphologically very similar, and represent a novel species closely related to *H. bacteriophora*. The nematode population HM was chosen as the type material to describe this newly discovered species.

### *Heterorhabditis casmirica* n. sp.

**Fig. 1:**
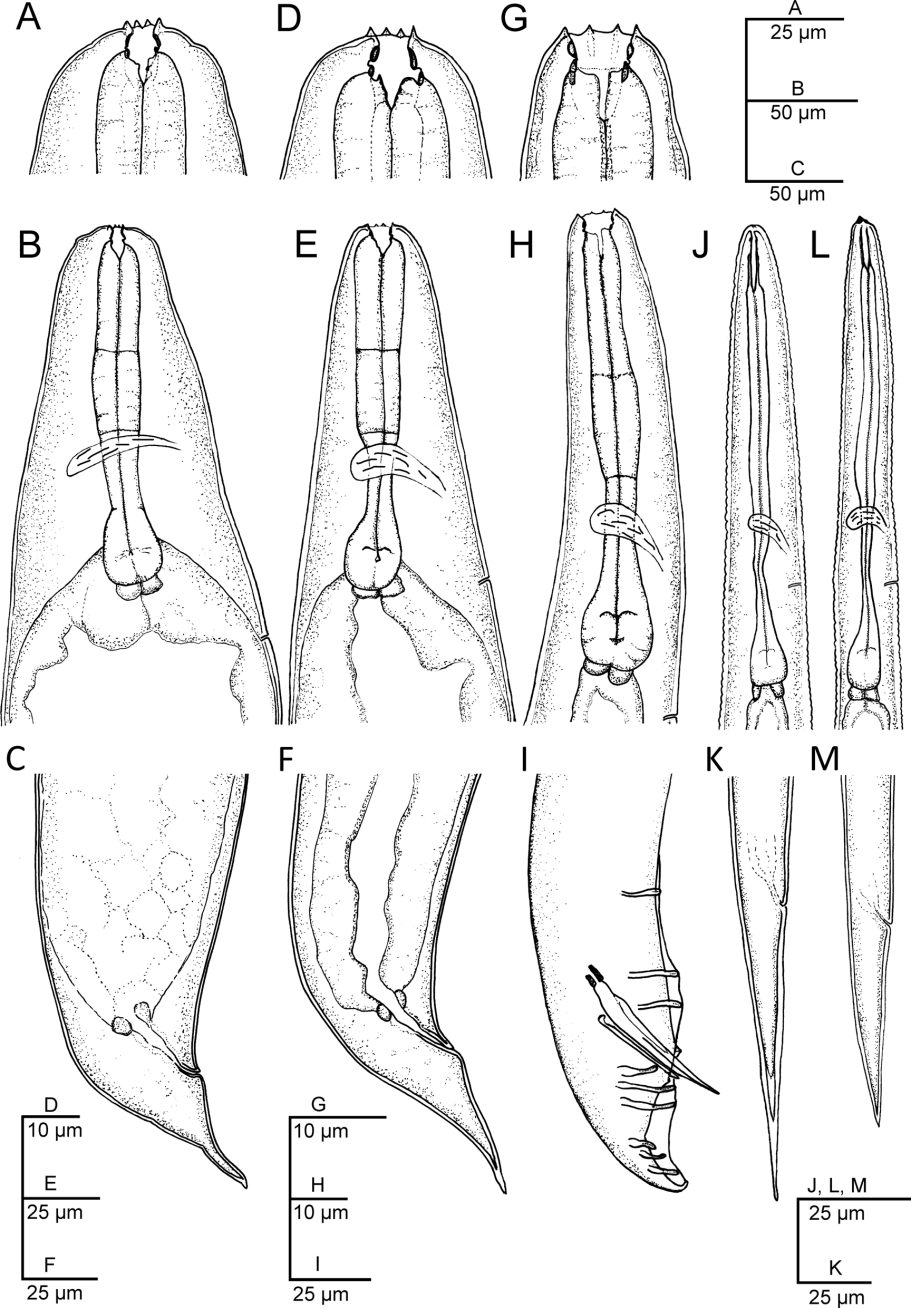
**a**–**m ***Heterorhabditis casmirica* n. sp. (drawings). **a**, **d**, **g** Anterior end of hermaphroditic female, amphimictic female and male, respectively. **b**, **e**, **h**, **j**, **l** Neck region of hermaphroditic female, amphimictic female, male, second-stage juvenile (J2) and third-stage juvenile (J3), respectively. **c**, **f**, **i**, **k**, **m** Posterior end of hermaphroditic female, amphimictic female, male, J2 and J3, respectively

**Fig. 2:**
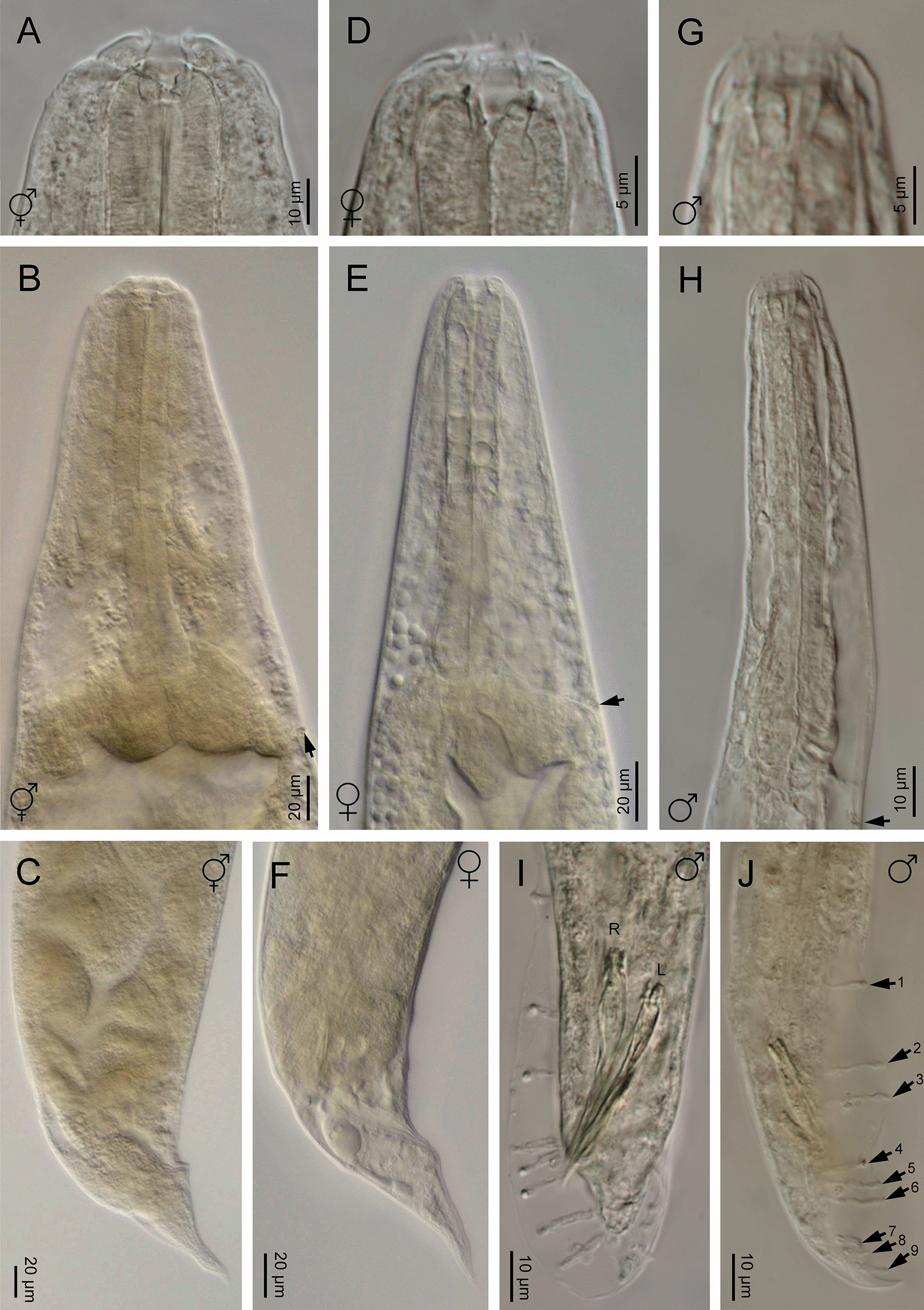
**a**–**j ***Heterorhabditis casmirica* n. sp. (light microscopy images). **a**, **d**, **g** Anterior end of hermaphroditic female, amphimictic female and male, respectively. **b**, **e**, **h** Neck region of hermaphroditic female, amphimictic female and male, respectively (arrowhead indicates the excretory pore). **c**, **f** Posterior end of hermaphroditic female and amphimictic female, respectively. **i**, **j** Posterior end of male at spicule and bursa levels, respectively [arrowhead indicates the genital papillae (GP)]

**Fig. 3:**
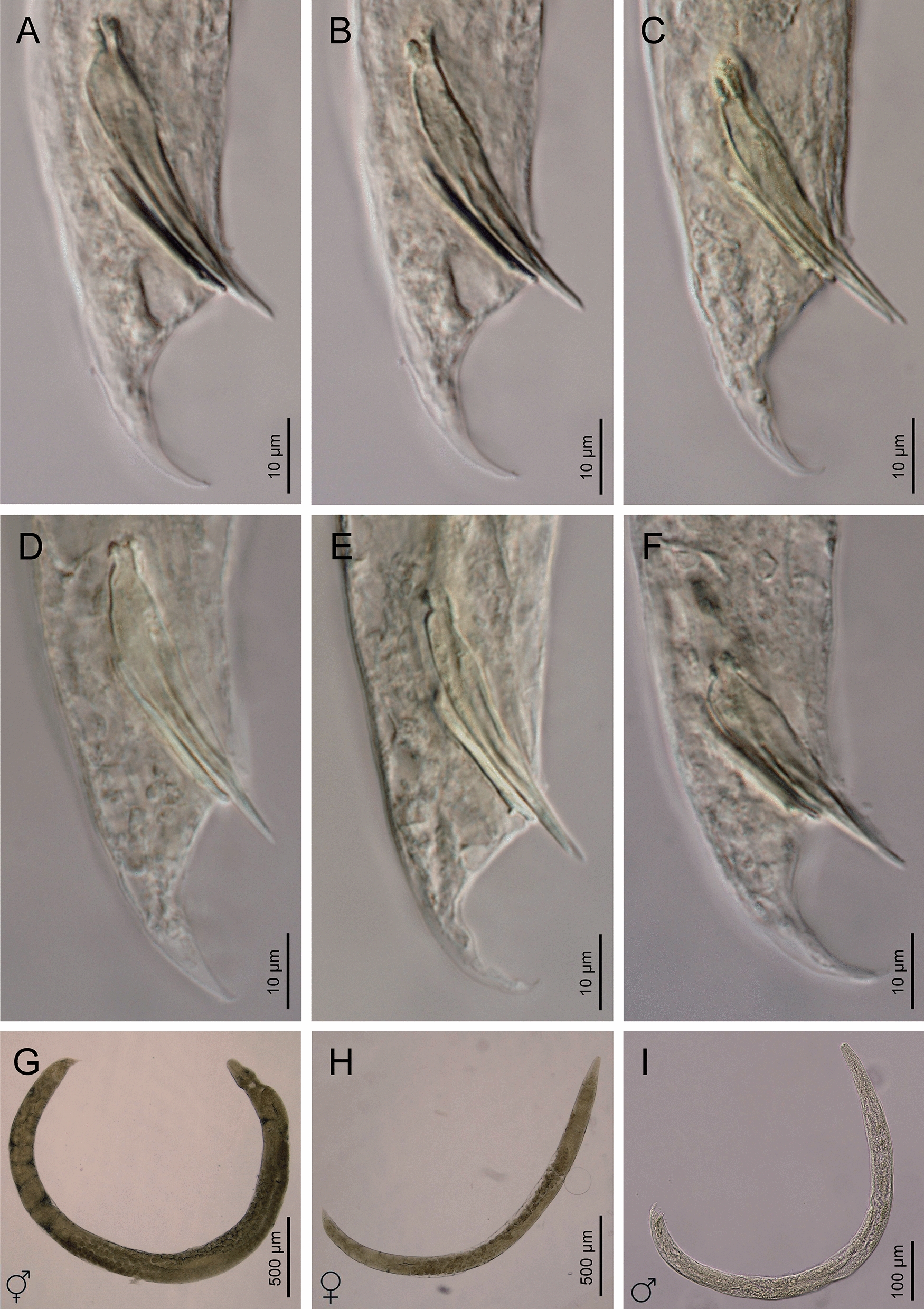
**a**–**i ***Heterorhabditis casmirica* n. sp. (light microscopy images). **a**–**f** Spicule and gubernaculum variability. **g** Hermaphroditic female. **h** Amphimictic female. **i** Male

**Fig. 4:**
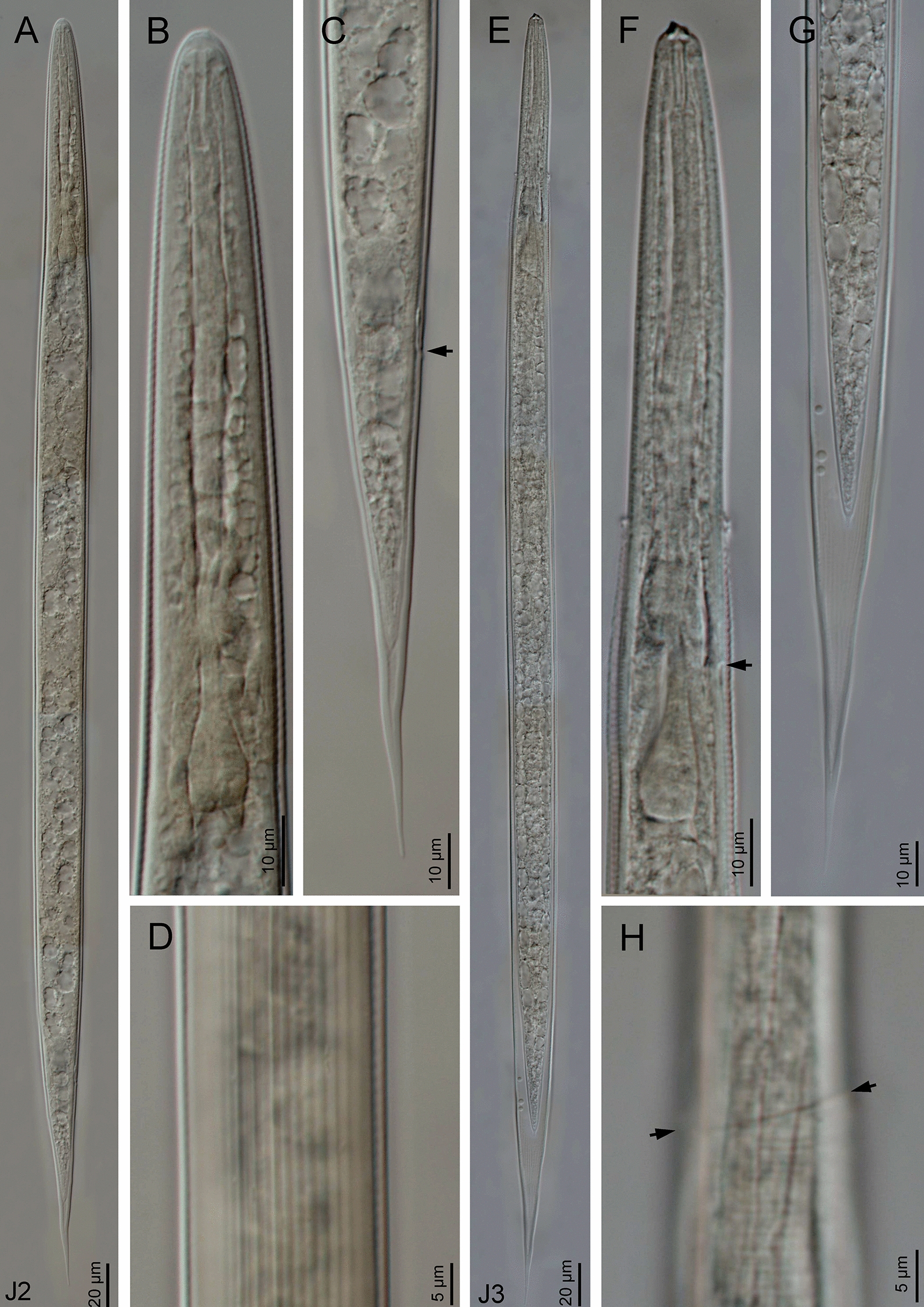
**a–h ***Heterorhabditis casmirica* n. sp. (light microscopy images). **a**, **e** Entire body of J2 and J3, respectively. **b**, **f** Neck region of J2 and J3, respectively (arrowhead indicates the excretory pore). **c**, **g** Posterior end of J2 and J3, respectively (arrowhead indicates the anus). **d**, **h** Cuticle of J2 and J3, respectively (arrowheads indicate the lateral field)

**Fig. 5:**
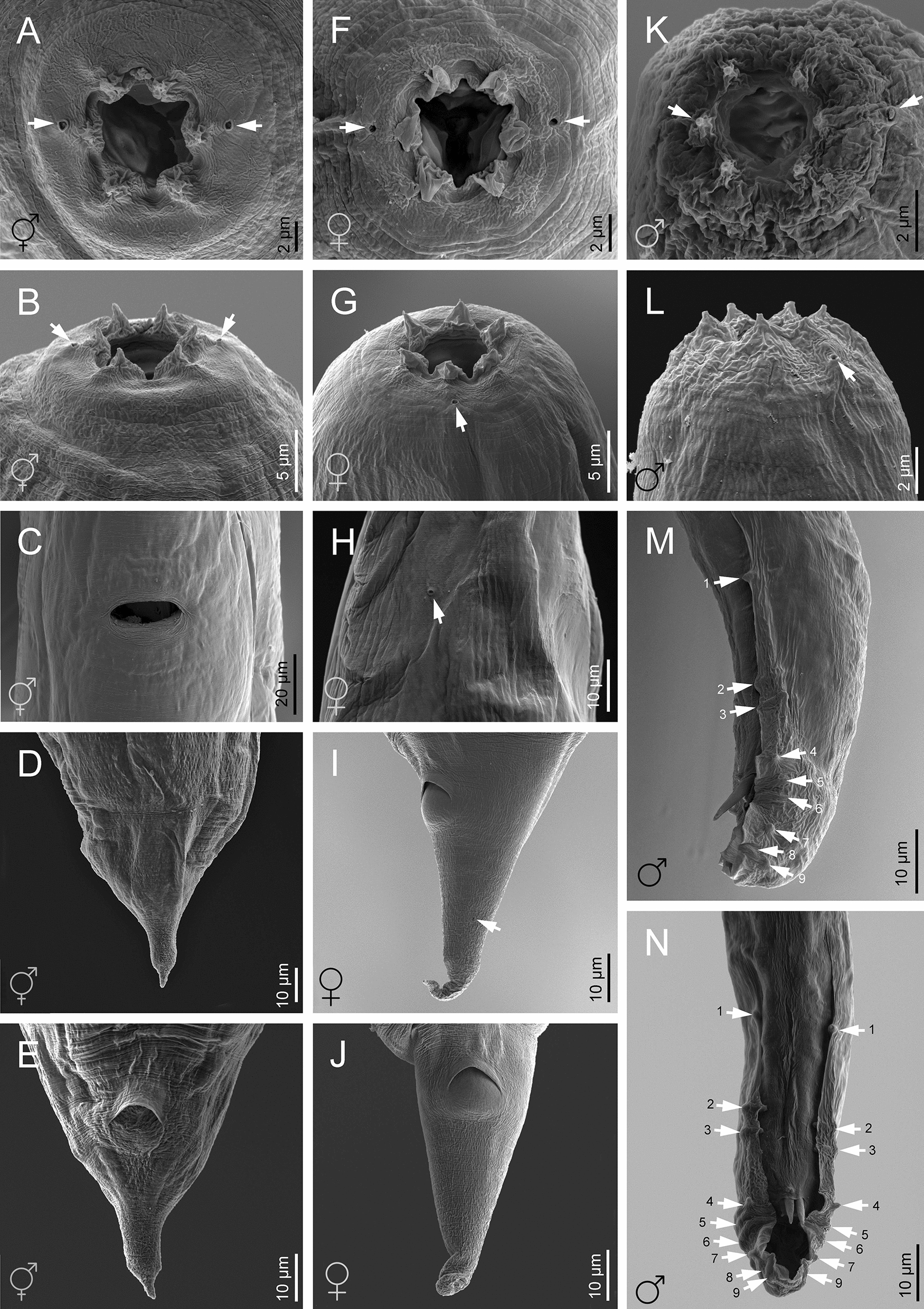
**a**–**n ***Heterorhabditis casmirica* n. sp. (scanning electron microscopy images). **a**, **f**, **k** Lip region (frontal view) in hermaphroditic female, amphimictic female and male, respectively (arrowheads indicate the amphids). **b**, **g**, **l** Lip region in hermaphroditic female (ventral view), amphimictic female (lateral view) and male (sublateral view), respectively (arrowheads indicate the amphids). **c** Vulva of hermaphroditic female. **d**, **i** Tail (lateral view) in hermaphroditic female and amphimictic female, respectively (arrowheads indicate the phasmid). **e**, **j** Tail (ventral view) in hermaphroditic female and amphimictic female, respectively. **h** Excretory pore (arrowhead) of amphimictic female. **m**, **n** Male posterior end (lateral and ventral views, respectively) (arrowheads indicate the bursal papillae)

**Fig. 6:**
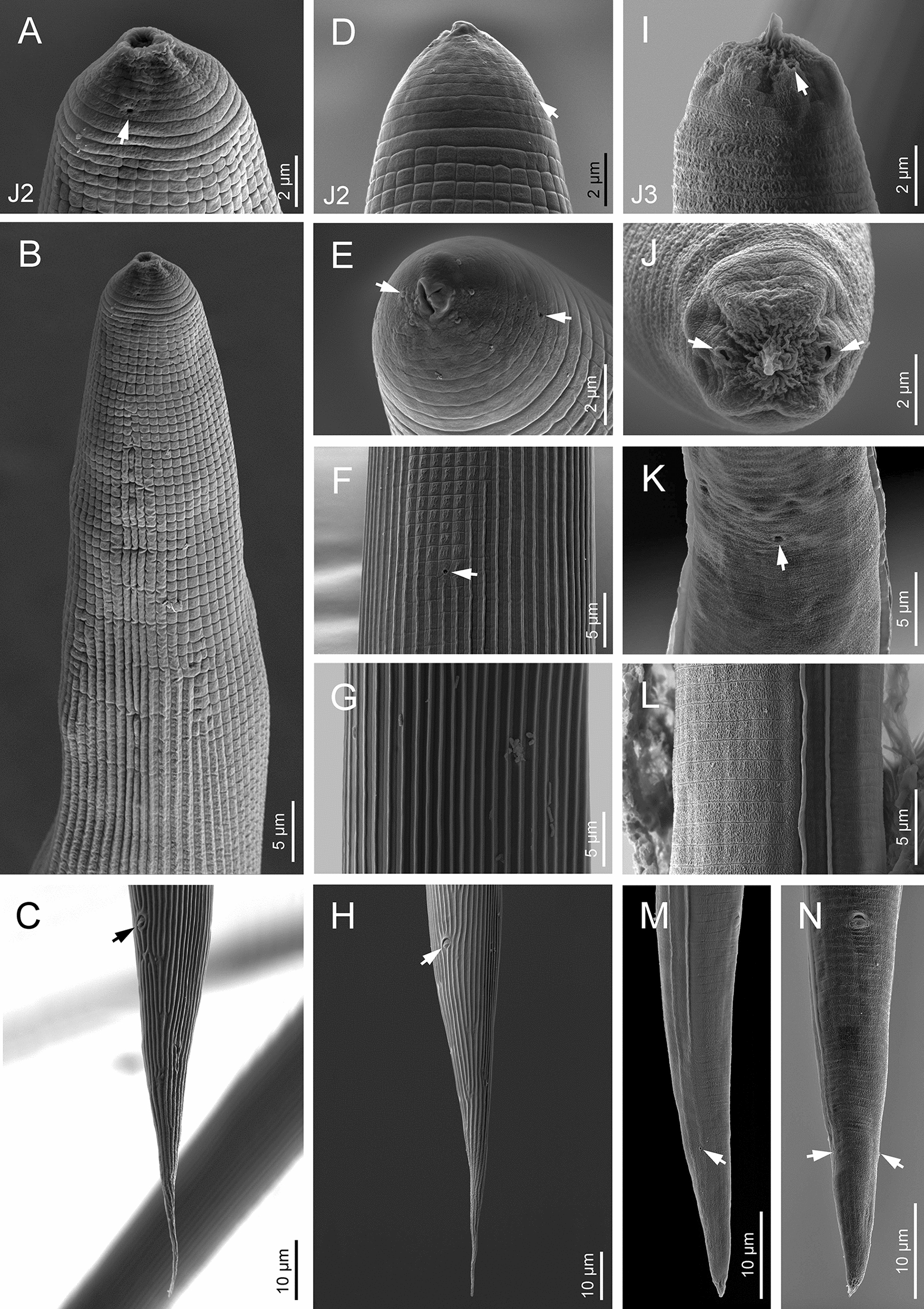
**a**–**n ***Heterorhabditis casmirica* n. sp. (scanning electron microscopy images). **a**, **d**, **e** Lip region of J2 (lateral, dorsal and frontal views, respectively) (arrowheads indicate the amphids). **b** Neck region of J2. **c**, **h** Tail of J2 (lateral and ventral views, respectively). **f** Excretory pore (arrowhead) of J2. **g** Cuticle at the mid-body region of J2. **i**, **j** Lip region of J3 (lateral and frontal views, respectively) (arrowheads indicate the amphids). **k** Excretory pore (arrowhead) of J3.** l** Lateral field of J3. **m**, **n** Tail of J3 (lateral and ventral views, respectively) (arrowheads indicate the phasmids)

Morphological and morphometric characteristics of *H. casmirica* n. sp are presented in Figs 1, 2, 3, 4, 5, 6 and Tables 1, 2, 3, 4, 5.

**Table 1:** Morphometrics of the infective juvenile (*IJ*) and adult generations of *Heterorhabditis casmirica* n. sp. (population HM)

Characters	Male (holotype)	Males (paratypes)	Hermaphrodites (paratypes)	Females (paratypes)	IJs (paratypes)
n	1	20	20	20	20
Body length (L)	875.5	754 ± 80 (608–914)	3466 ± 392 (2851–4219)	1432 ± 172 (1273–1990)	563 ± 22 (512–599)
*a* (L/BD)	18.3	18 ± 2.8 (15–24)	24 ± 3.2 (19–27)	20 ± 1.9 (14–25)	23 ± 1.1 (20–25)
*b* (L/NL)	7.6	7.3 ± 0.4 (6.4–8.2)	24 ± 4.6 (16–39)	11 ± 0.8 (10–13)	4.5 ± 0.2 (4.0–5.2)
*c* (*L*/*T*)	30.5	29 ± 3.8 (24–35)	72 ± 32 (56–84)	19 ± 3.2 (16–31)	5.4 ± 0.5 (4.7–6.4)
*c*′ (T/ABD)	1.3	1.2 ± 0.2 (1.1–1.6)	1.8 ± 0.2 (1.4–2.2)	1.9 ± 0.1 (1.6–2.5)	6.0 ± 0.7 (5.1–8.0)
*V*% (VA/L × 100)	–^a^	–	52 ± 2.7 (46–57)	49 ± 2.1 (45–52)	–
Maximum body diameter (BD)	46.2	36 ± 7.4 (24–48)	230 ± 50 (140–341)	91 ± 17 (70–135)	20 ± 1.0 (17–24)
Excretory pore—anterior end (EP)	83.9	114 ± 4.3 (102–120)	194 ± 8.5 (180–211)	146 ± 7.5 (135–157)	106 ± 6 (98–129)
Width at excretory pore (WEP)	27.8	24 ± 3.2 (19–29)	93 ± 12.2 (71–108)	49 ± 5.6 (41–61)	18 ± 1.3 (14–24)
Nerve ring—anterior end (NR)	60.3	72 ± 6.1 (58–80)	86 ± 9.1 (77–100)	95 ± 6.3 (84–111)	89 ± 4.3 (79–94)
Pharynx length (PL)	98.5	108 ± 7.8 (96–114)	186 ± 9.0 (168–202)	137 ± 7.6 (126–149)	116 ± 5.1 (99–127)
Neck length (NL)	111.7	112 ± 8.2 (100–118)	191 ± 9.5 (174–207)	141 ± 8.3 (132–156)	119 ± 5.4 (104–132)
Bulb length (BL)	16.9	18 ± 2.2 (14–23)	34 ± 2.8 (28–40)	28 ± 2.2 (25–33)	15 ± 1.3 (12–19)
Bulb width (BW)	13.7	12 ± 1.3 (10–15)	26 ± 1.4 (22–30)	20 ± 1.6 (18–23)	8.5 ± 1.6 (6.7–13)
Tail length (*T*)	28.6	25 ± 4.5 (16–32)	82 ± 12 (72–114)	75 ± 5.9 (64–83)	99 ± 7.8 (85–115)
Anal body diameter (ABD)	22.5	18 ± 3.1 (14–25)	44 ± 4.4 (36–56)	26 ± 2.0 (22–30)	15 ± 1.7 (11–20)
Spicule length (SL)	43.5	41 ± 2.9 (38–48)	–	–	–
Gubernaculum length (GL)	23.8	22 ± 2.0 (18–26)	–	–	–
*D*% (EP/NL × 100)	96.4	102 ± 2.3 (99–107)	102 ± 4.8 (94–120)	103 ± 4.0 (99–116)	90 ± 3.8 (83–97)
*E*% (EP/T × 100)	295.3	325 ± 31 (268–394)	236 ± 23 (205–292)	177 ± 15 (156–209)	115 ± 9.1 (93–125)
SW% (SL/ABD × 100)	193.2	212 ± 27 (160–252)	–	–	–
GS% (GL/SL × 100)	54.6	53 ± 4.3 (45–63)	–	–	–
Body width at vulva	–	–	182 ± 17 (152–218)	90 ± 17 (70–132)	–
Vulva—anterior end (VA)	–	–	1545 ± 152 (1312–1883)	708 ± 80 (631–918)	–
Vulva—posterior end (VP)	–	–	1918 ± 182 (1466–2429)	726 107 (617–1076)	–

All data, with the exception of *n*, ratios and percentages, are given in micrometers, and are shown as the mean ± SD (range)

^a^Dashes indicate that these characters are absent in these generations

**Table 2:** Comparative morphometrics of *Heterorhabditis* IJs

Species	*L*	BD	EP	NR	NL	*T*	*a*	*b*	*c*	*c′*	*D*%	*E*%	Country	References
*Heterorhabditis amazonensis*	567–612	20–24	89–115	76–93	107–132	98–115	24–29	4.4–5.5	5.1–6.1	7.3^b^	83–92	89–109	Brazil	Andaló et al. [65]
*Heterorhabditis atacamensis*	578–666	19–26	101–126	79–101	124–144	94–107	25–31	4.8–5.7	5.7–7.1	5.7^b^	79–94	149–182	Chile	Edgington et al. [66]
*Heterorhabditis bacteriophora*	512–671	18–31	87–110	72–93	100–139	83–112	17–30	4.0–5.1	5.7–7.0	6.0^b^	76–92	103–130	Australia	Poinar [62]
*Heterorhabditis baujardi*	497–595	18–22	91–103	75–86	107–120	83–97	26–30	4.5–5.1	6.0–6.7	7.2^b^	78–88	98–114	Vietnam	Phan et al. [67]
*Heterorhabditis beicherriana*	566–687	21–25	100–122	85–106	118–146	86–111	24–29	4.2–4.9	5.9–6.8	6.0–7.4	80–93	103–121	China	Li et al. [63]
*Heterorhabditis casmirica* n. sp.	*512–599*	*17–24*	*98–129*	*79–94*	*114–138*	*85–115*	*20–25*	*4.0–5.2*	*4.7–6.4*	*5.1–8.0*	*83–97*	*93–136*	*India*	*This study*
*Heterorhabditis downesi*	588–692	15–22	96–128	96–105	126–141	62–74	29–42	4.4–5.3	8.5–10.5	4.4^b^	76–96	160–180	Ireland	Stock et al. [68]
*Heterorhabditis egyptii*	484–515	18–23	81–94	78–100	100–119	53–75	20–27	4.2–5.2	6.8–9.1	6.9^b^	74–82	100–170	Egypt	Abd-Elgawad & Ameen [18]
*Heterorhabditis floridensis*	554–609	19–23	101–122	68–107	123–142	91–113	25–32	3.9–4.9	5.3–6.6	7.2^b^	71–90	95–134	USA	Nguyen et al. [69]
*Heterorhabditis georgiana*	547–651	17–26	97–113	74–94	110–139	86–108	23–34	4.1–5.3	5.5–6.9	6.8^b^	70–93	106	USA	Nguyen et al. [64]
*Heterorhabditis hambletoni*	–^a^	–	–	–	–	–	–	–	–	–	–	–	Brazil	Pereira [19]
*Heterorhabditis indica*	479–573	19–22	88–107	72–85	109–123	93–109	25–27	4.3–4.8	4.5–5.6	–	79–90	83–103	India	Poinar et al. [20]
*Heterorhabditis marelatus*	588–700	24–32	81–113	83–113	121–139	99–117	21–29	4.7–5.4	5.5–6.6	3.0^b^	60–86	89–110	USA	Liu & Berry [70]
*Heterorhabditis megidis*	736–800	27–32	123–142	104–115	147–160	112–128	23–38	4.6–5.9	6.1–6.9	6.3^b^	81–91	103–120	USA	Poinar et al. [71]
*Heterorhabditis mexicana*	530–620	20–24	83–109	74–88	104–142	91–106	24–28	4.2–5.1	5.5–6.3	8.3^b^	72–86	87–111	Mexico	Nguyen et al. [72]
*Heterorhabditis noenieputensis*	484–578	21–25	88–105	69–96	79–115	78–95	21–27	4.3–5.2	5.5–6.8	3.4–4.3	81–95	99–125	S. Africa	Malan et al. [73]
*Heterorhabditis ruandica*	496–591	18–27	70–89	52–64	75–102	49–64	20–29	5.1–6.6	7.6–8.6	3.4–5.8	65–98	99–157	Rwanda	Machado et al. [17]
*Heterorhabditis safricana*	550–676	19–23	103–122	86–101	125–141	86–108	25–32	3.9–4.9	5.4–7.5	8.7^b^	80–90	99–133	S. Africa	Malan et al. [74]
*Heterorhabditis taysearae*	332–499	17–23	74–113	58–87	96–130	44–70	18–27	3.4–4.2	6.5–8.7	3.7^b^	71–96	110–230	Egypt	Shamseldean et al. [75]
*Heterorhabditis zacatecana*	493–578	23–27	72–99	69–72	78–99	52–63	19–24	5.3–7.2	8.2–10	4.3–6.7	72–122	128–184	Mexico	Machado et al. [17]
*Heterorhabditis zealandica*	570–740	22–30	94–123	90–107	135–147	87–119	25	4.9	6.7	–	73–92	103–109	New Zealand	Poinar [76]

All data, with the exception of ratios and percentages, are given in micrometers, and are shown as the mean ± SD (range). Data for *H. casmirica* n. sp. are in italic. For abbreviations, see Table 1

^a^Dashes indicate that the data are not provided in the original publication

^b^Calculated from the drawings provided in the original publication

**Table 3:** Comparative morphometrics of *Heterorhabditis* adult males

Species	*L*	BD	EP	NR	NL	*T*	SL	GL	*a*	*b*	*c*	*c′*	SW%	GS%	*D*%
*Heterorhabditis amazonensis*	692–826	36–43	96–116	71–88	97–114	29–41	35–45	19–23	18.7^b^	7.7^c^	27.5^c^	1.3^c^	120–187	44–56	95–109
*Heterorhabditis atacamensis*	842–1025	42–55	116–149	69–93	99–119	24–36	40–49	17–22	19.7^b^	9.6^c^	29.3^c^	1.5^c^	179–249	38–51	108–126
*Heterorhabditis bacteriophora*	780–960	38–46	114–130	65–81	99–105	22–36	36–44	18–25	20.8^b^	9.1^b^	34.3^b^	1.8^b^	174	50	117
*Heterorhabditis baujardi*	818–970	45–53	71–93	54–77	105–132	28–38	33–45	18–22	16–22	6.4–8.8	24–33	1.5^c^	138–208	44–61	79^c^
*Heterorhabditis beicherriana*	889–1192	51–73	130–157	81–108	116–143	32–45	40–49	22–27	15–23	7.2–10	22–34	1.3–2.3	153–208	48–59	102–120
*Heterorhabditis casmirica* n. sp.	*608–914*	*24–48*	*102–120*	*58–80*	*100–118*	*16–32*	*38–48*	*18–26*	*15–24*	*6.4–8.2*	*24–35*	*1.1–1.6*	*160–252*	*45–63*	*99–107*
*Heterorhabditis downesi*	699–876	33–40	86–91	62–78	97–106	29–34	41–47	17–19	26.6^b^	8.8^c^	27.4^c^	1.4^c^	170–220	36–47	90
*Heterorhabditis egyptii*	594–848	31–56	80–97	56–84	96–109	23–34	25–50	16–22	17.1^b^	6.6^c^	19.5^c^	1.5^b^	120–220	40–65	84–91
*Heterorhabditis floridensis*	785–294	43–50	104–128	73–90	97–111	29–40	36–46	17–30	19.9^b^	7.9^c^	24.1^c^	1.4^c^	133–209	47–65	112
*Heterorhabditis georgiana*	721–913	43–55	101–145	72–93	100–122	29–41	41–49	20–28	16.5^b^	7.7^c^	26.1^c^	1.4^c^	150–200	51–64	100–122
*Heterorhabditis hambletoni*	510–800	38–60	80–100	80–90	–	–	–	–	–	–	–	–	–	–	–
*Heterorhabditis indica*	573–788	35–46	109–138	72–85	93–109	24–32	35–48	18–23	17.6^b^	6.7^c^	23.0^c^	1.1^c^	187	49	121
*Heterorhabditis marelatus*	805–1046	48–56	110–168	61–95	99–123	24–38	41–49	18–22	15.5^b^	7.8^c^	30.0^c^	1.1^c^	196	36–50	113^c^
*Heterorhabditis megidis*	800–1100	44–50	139–176	96–112	122–134	35–43	46–54	17–24	18–22	7–9	23–31	1.6^b^	188	43	122
*Heterorhabditis mexicana*	614–801	38–47	108–145	61–83	89–108	21–36	30–47	18–32	21.7^b^	6.8^c^	27.6^c^	1.1^c^	130–196	43–70	114–149
*Heterorhabditis noenieputensis*	530–775	34–46	75–102	64–75	88–106	21–32	37–49	17–24	14–18	5.6–7.9	21–33	1.1–1.7	202–301	38–56	81–108
*Heterorhabditis ruandica*	652–863	40–51	61–109	56–74	84–117	21–29	34–50	16–23	15–21	5.8–9.7	23–36	0.6–1.7	150–306	35–57	61–97
*Heterorhabditis safricana*	777–1009	40–58	104–147	52–61	105–126	27–49	35–54	19–27	20.1^b^	7.9^c^	43.0^c^	1.5^b^	130–259	43–62	92–133
*Heterorhabditis taysearae*	648–736	38–48	78–120	54–88	85–123	20–29	30–42	12–21	15.1^b^	6.5^c^	14.0^c^	1.3^c^	156	46	88
*Heterorhabditis zacatecana*	811–914	41–56	77–109	60–78	71–108	21–33	38–55	15–25	15–25	7.6–12	26–43	1.2–2.5	170–320	40–60	78–134
*Heterorhabditis zealandica*	848–1044	36–45	130–150	–	110–128	30–41	48–55	19–25	–	–	–	1.7^b^	246	44	118

All data, with the exception of ratios and percentages, are given in micrometers, and are shown as the mean ± SD (range). Data for *H. casmirica* n. sp. are in italic. For abbreviations, see Table 1

^a^Dashes indicate that the data are not provided in the original publication

^b^Calculated from the drawings provided in the original publication

^c^Calculated from other measurements provided in the original publication

**Table 4:** Comparative morphometrics of *Heterorhabditis* hermaphroditic females

Species	*L*	BD	EP	NR	NL	*T*	*a*	*b*	*c*	*c′*	*V*%	ABD	*D*%
*Heterorhabditis amazonensis*	3517–5587	220–316	184–238	128–171	180–225	104–154	–	–	–	2.3^b^	42–47	59–83	103^b^
*Heterorhabditis atacamensis*	1791–2904	88–122	165–206	101–132	174–200	72–112	–	–	–	2.7^b^	39–48	30–46	90–114
*Heterorhabditis bacteriophora*	3630–4390	160–180	189–217	121–130	189–205	81–93	–	–	–	–	41–47	40–53	106
*Heterorhabditis baujardi*	3135–4170	180–240	156–192	119–147	186–206	66–114	15–19	16–21	36–50	2.0^b^	43–48	47–63	88^b^
*Heterorhabditis beicherriana*	3671–5543	198–374	165–297	135–243	192–343	68–130	13–20	13–25	34–62	1.0–2.3	41–49	51–92	76–94
*Heterorhabditis casmirica* n. sp.	*2851–4219*	*140–341*	*180–211*	*77–100*	*174–207*	*72–114*	*19–27*	*16–39*	*56–84*	*1.4–2.2*	*46–57*	*36–56*	*94–120*
*Heterorhabditis downesi*	3030–5051	183–291	200–254	175–230	230–244	60–70	–	–	–	1.1^b^	50–55	57–65	117^b^
*Heterorhabditis egyptii*	2100–3100	107–164	154–205	101–147	144–192	83–115	–	–	–	2.7^b^	46–59	33–51	104^b^
*Heterorhabditis floridensis*	3731–5865	217–331	211–301	169–271	271–391	84–126	–	–	–	2.5^b^	44–49	42–78	104^b^
*Heterorhabditis georgiana*	3232–4928	157–267	200–277	143–217	132–271	65–96	–	–	–	1.2^b^	44–55	42.6^b^	–
*Heterorhabditis hambletoni*	–	–	–	–	–	–	–	–	–	–	–	–	–
*Heterorhabditis indica*	2300–3100	107–145	163–187	104–123	163–179	72–110	–	–	–	–	45–50	38–51	–
*Heterorhabditis marelatus*	3000–4500	161–233	212–287	133–182	190–244	75–101	–	–	–	1.3^b^	45–50	20–28	109^b^
*Heterorhabditis megidis*	2400–4900	120–133	193–270	139–178	106–269	95–124	14–24	12–21	23–49	–	45–50	36–86	–
*Heterorhabditis mexicana*	2440–4606	135–267	103–201	114–171	168–221	94–170	–	–	–	2.6^b^	30–58	40–46	90^b^
*Heterorhabditis noenieputensis*	2987–5498	168–289	152–209	112–152	166–220	79–120	14–23	18–28	37–58	1.7–3.4	39–47	26–56	77–112
*Heterorhabditis ruandica*	2907–4123	209–274	106–153	78–108	134–159	63–98	12–16	21–27	34–51	1.7–2.6	45–55	29–51	67–103
*Heterorhabditis safricana*	3373–4073	127–188	210–267	121–163	199–236	64–91	–	–	–	–	43–46	40–54	98–119
*Heterorhabditis taysearae*	2200–2800	116–170	137–182	83–120	161–200	72–100	–	–	–	–	40–64	41–67	–
*Heterorhabditis zacatecana*	4408–6179	235–385	108–190	96–169	174–231	63–87	13–20	20–34	52–90	1.2–2.4	36–57	34–58	55–95
*Heterorhabditis zealandica*	–^a^	–	–	–	–	–	–	–	–	–	–	–	–

All data, with the exception of ratios and percentages, are given in micrometers, and are shown as the mean ± SD (range). Data for *H. casmirica* n. sp. are in italic. For abbreviations, see Table 1

^a^Dashes indicate that the data are not provided in the original publication

^b^Calculated from the drawings provided in the original publication

**Table 5:** Comparative morphometrics of *Heterorhabditis* amphimictic females

Species	*L*	BD	EP	NR	NL	*T*	*a*	*b*	*c*	*c′*	*V*	ABD	*D*%
*Heterorhabditis amazonensis*	1279–2070	70–122	103–126	68–100	119–142	25–38	–	–	–	2.4^b^	46–50	25–38	–
*Heterorhabditis atacamensis*	1754–2628	86–129	154–182	79–119	129–167	80–108	–	–	–	3.8^b^	43–49	24–33	100–113
*Heterorhabditis bacteriophora*	3180–3850	160–220	174–214	93–118	155–183	71–93	21.4^b^	18.8	41.5^b^	3.1^b^	42–53	22–31	114
*Heterorhabditis baujardi*	1335–2130	90–150	104–149	75–122	131–185	68–89	12–16	10–12	19–32	–	46–51	27–41	–
*Heterorhabditis beicherriana*	1581–3026	125–218	95–165	59–138	105–186	68–105	10–18	10–23	19–34	1.6–2.4	41–49	35–81	88–98
*Heterorhabditis casmirica* n. sp.	*1273–1990*	*73–150*	*135–157*	*84–111*	*132–156*	*64–83*	*14–15*	*10–13*	*16–31*	*1.6–2.5*	*45–52*	*22–30*	*99–116*
*Heterorhabditis downesi*	1231–2728	74–131	99–126	117–151	111–155	70–122	–	–	–	2.5^b^	47–60	25–38	–
*Heterorhabditis egyptii*	1050–1420	56–84	69–106	69–94	106–125	56–78	17.5^c^	14.4^c^	22.2^c^	3.1^c^	44–51	19–27	78^c^
*Heterorhabditis floridensis*	2054–2548	120–156	110–168	86–122	126–178	69–87	–	–	–	–	44–50	32–42	–
*Heterorhabditis georgiana*	1640–2779	101–188	111–177	96–162	136–219	62–88	–	–	–	1.5^b^	46–53	42^b^	–
*Heterorhabditis hambletoni*	600–1200	70–100	80–90	70–80	–	–	–	–	–	–	50–58^c^	–	–
*Heterorhabditis indica*	1200–1800	76–113	118–138	88–96	120–139	66–88	–	–	–	–	40–53	22–32	–
*Heterorhabditis marelatus*	1600–2600	113–177	139–178	79–119	129–164	55–81	–	–	–	1.3^b^	45–50	29–48	110^b^
*Heterorhabditis megidis*	1500–2500	95–140	158–206	105–120	155–168	70–101	15–19	10–16	18–32	2.6^b^	47–51	25–38	119^b^
*Heterorhabditis mexicana*	1144–2108	65–123	114–148	76–103	121–150	76–106	–	–	–	–	44–51	21–36	–
*Heterorhabditis noenieputensis*	1075–1697	76–129	102–125	73–90	115–132	63–75	13–17	9–14	17–24	2.3–3.1	40–53	22–32	83–104
*Heterorhabditis ruandica*	1131–1608	68–83	92–129	69–97	107–132	62–88	15–20	9.0–14	16–24	1.9–3.6	41–51	18–34	74–104
*Heterorhabditis safricana*	1679–2937	102–229	151–196	87–139	148–180	55–111	–	–	–	1.3^b^	45–50	25–72	97–120
*Heterorhabditis taysearae*	830–1400	42–96	120–166	76–109	129–179	62–80	–	–	–	4.0^b^	44–73	19–28	82^b^
*Heterorhabditis zacatecana*	1954–2798	160–228	100–133	71–96	112–148	45–75	11–15	16–21	31–63	1.3–2.0	43–61	31–41	80–111
*Heterorhabditis zealandica*	–^a^	–	–	–	–	–	–	–	–	–	–	–	–

All data, with the exception of ratios and percentages, are given in micrometers, and are shown as the mean ± SD (range). Data for *H. casmirica* n. sp. are in italic. For abbreviations, see Table 1

^a^Dashes indicate that the data are not provided in the original publication

^b^Calculated from the drawings provided in the original publication

^c^Calculated from other measurements provided in the original publication

#### Hermaphroditic females

Hermaphroditic female body C-shaped when heat relaxed, body robust, always containing many juveniles, in some specimens a few eggs were visible. Cuticle almost smooth, about 0.8 to 1.6 µm thick. Lateral fields and phasmids not distinguishable under LM. Anterior end tapering anteriorly. Labial region with six prominent lips, each with a terminal conoid labial papilla. Cephalic papillae not observed with LM. Amphidial apertures pore-like. Stoma rhabditoid type, 1.1–1.7 times the lip region width, with a short cheilostom with a hardly visible refringent rounded cheilorhabdia, gymnostom with refringent bar-like rhabdia, well-developed, and funnel-shaped stegostom surrounded by the pharyngeal collar and bearing minute rhabdia. Pharynx with sub-cylindrical procorpus, slightly swollen metacorpus, robust isthmus, and poorly developed, spheroid basal bulb with inconspicuous valves. Nerve ring surrounding the isthmus, at 55–74% of neck length. Excretory pore at basal bulb level or intestine level, at 94–120% of neck length. Cardia conoid. Reproductive system didelphic–amphidelphic. Ovaries well developed, reflexed. Oviducts poorly differentiated. Uteri with numerous embryonated eggs. Vagina short. Vulva a transverse slit, with smooth top and scarcely prominent lips, close to mid-body. Rectum slender, about 0.9–1.4 times the anal body diameter. Anal region swelling posteriorly. Tail conoid with narrower pointed terminus, lacking a mucron. Phasmids inconspicuous.

#### Amphimictic females

Body arcuate with general morphology similar to that of hermaphroditic females. Body tapering toward anterior end; labial papillae acute and prominent. Reproductive system didelphic–amphidelphic with ovaries well developed, reflexed, oviducts and uteri poorly visible, vagina very short, and vulva small with a transverse slit opening. Rectum slightly longer than that of hermaphroditic females, about 1.7–1.9 times longer than the anal body diameter. Anal lips usually prominent. Tail conoid longer than that of hermaphroditic females, with acute tip lacking a mucron. Phasmids very small, located at 50–62% of tail length.

#### Males

Body curved ventrally (open C-shape) or sometimes straight when heat relaxed. Anterior end truncate. Lip region with six scarcely separated lips and six conoid liplets at oral margin; six labial papillae at liplet tips and four cephalic papillae at the base of the dorsal and ventral lips. Amphidial aperture pore-like, just posterior to the lateral lips. Stoma 0.8–1.4 times the lip region width, with short cheilostom and hardly visible refringent rounded cheilorhabdia, short gymnostom with refringent bar-like rhabdia, and long, funnel-shaped stegostom surrounded by the pharyngeal collar and bearing minute rhabdia. Pharynx with subcylindrical procorpus, scarcely swollen metacorpus, isthmus robust and slightly narrower than metacorpus, and basal bulb poorly developed, spheroid, with poorly developed valvular apparatus. Nerve ring located surrounding isthmus, at 55–69% of neck length. Excretory pore located at basal bulb or intestine level, at 99–107% of neck length. Cardia conoid, protruding into intestine. Intestine without differentiation although with narrower walls at anterior end. Reproductive system monorchid, with testis anteriorly reflexed and vas deferens well developed. Spicules well developed, separate, with small, almost quadrangular manubrium with very refringent dorsal and ventral walls, frequently smaller at the left spicule, calamus developed, and almost straight lamina with acute tip, poorly developed dorsal hump, and ventral velum slightly developed. Gubernaculum robust, straight or slightly curved ventrally, 40–63% of spicule length, with manubrium visibly hook-like. Tail conoid with acute tip, ventrally curved posteriorly, flanked by the bursa. Bursa peloderan bearing nine pairs of bursal papillae 1 + 2/3 + 3: three precloacal and six postcloacal, with genital papilla 4 (GP4) and genital papilla 7 (GP7) open outside.

#### Infective sheathed juveniles (third-stage juvenile ensheathed in cuticle of second-stage juvenile)

Body straight when heat relaxed. Sheath (second-stage cuticle) present. Cuticle with longitudinal ridges except for the anterior part of the body, with annuli at the lip region and with tessellate pattern posterior to the lip region. Lip region lacking differentiated lips, bearing six labial papillae and cephalic papillae not visible. Amphidial aperture pore-like, having a cuticular dimple-like structure at its anterior part. Oral opening triradiate, closed. Stoma tubular, about twice as wide as the lip region. Pharynx slender, with corpus subcylindrical, isthmus narrower and slender, and basal bulb pyriform without developed valves. Nerve ring surrounding the isthmus, at 64–76% of neck length. Excretory pore at isthmus level, at 81–94% of neck length. Hemizonid clearly visible. Cardia conoid, surrounded by the intestinal tissue. Bacterial pouch not visible. Lateral fields not well differentiated from cuticle. Rectum narrow, not clearly discernible. Anus not well developed. Tail conoid-elongate with finely rounded terminus, without mucron. Terminal hyaline part 30–45% of tail length. Phasmids not visible.

#### Infective non-sheathed juveniles (third-stage juvenile)

Body with habitus straight when heat relaxed. Cuticle with transversal striae (annuli). Lateral field with two prominent longitudinal ridges. Lip region rounded, lacking differentiated lips, and labial and cephalic papillae not visible. Amphidial apertures oval. Oral opening rounded, closed, bearing a small dorsal tooth. Stoma, pharynx, nerve ring and excretory pore location similar to the sheathed stage. Hemizonid well developed. Cardia conoid, surrounded by intestinal tissue. Rectum narrow and hardly visible. Anus closed. Tail conoid with refringent acute tip without mucron. Phasmids very small, located at posterior part of tail.

### Diagnosis of *H. casmirica* n. sp.

*Heterorhabditis casmirica* n. sp. is characterized by having females and males with six conoid oral liplets, pore-like amphids and a robust pharynx, pharynx slender in juveniles, nerve ring surrounding the isthmus and excretory pore at basal bulb or intestine level in adults and at isthmus level in juveniles. Hermaphroditic females 2.8–4.2 mm long, with conoid tail (72–114 µm long, *c* = 56–84, *c*′ = 1.4–2.2) with narrower tip; amphimictic females 1.2–2.0 mm long, with conoid tail (64–83 µm long, *c* = 16–31, *c*′ = 1.6–2.5); males 0.6–0.9 mm long, with ventrally curved tail (16–32 µm long, *c* = 24–32, *c*′ = 1.1–1.6), bursa with nine bursal papillae, spicules 38–48 µm long with manubrium with refractive walls, frequently smaller at the left spicule, gubernaculum 18–26 µm long with hook-like manubrium; juvenile with a tubular stoma and narrow and slender pharynx, second-stage juvenile (J2) 0.4–0.5 µm long, with cuticle with longitudinal ridges and conoid-elongate tail with finely rounded tip, and third-stage juvenile (J3) 0.5–0.6 µm long, with transversal annuli, a lateral field with two longitudinal ridges, oral opening with dorsal tooth and conoid tail with refringent acute tip.

### Morphological relationships of *H. casmirica* n. sp. with other closely related species

*Heterorhabditis casmirica* n. sp. shares morphological similarities with *Heterorhabditis bacteriophora* [62], *Heterorhabditis beicherriana* [63], *Heterorhabditis egyptii* [18], *Heterorhabditis georgiana* [64], *Heterorhabditis ruandica* [17], and *Heterorhabditis zacatecana* [17]. However, several distinct morphological and morphometric characteristics can be used to differentiate *H. casmirica* n. sp. from these closely related species (Tables 2, 3, 4, 5).

IJs of *H. casmirica* n. sp. can be differentiated from those of *H. bacteriophora* by differences in the* c* ratio (4.7–6.4 vs. 5.7–7.0), the presence of a bacterial sac (invisible vs. visible in the ventricular portion of the intestine), and size of phasmids (very small at the posterior part of the tail vs. inconspicuous). Compared to *H. beicherriana* IJs, those of *H. casmirica* n. sp. differ in the shape of amphidial apertures (oval vs. inconspicuous), the position of the excretory pore (at isthmus level vs. at the beginning of the basal bulb), visibility of the bacterial sac (invisible vs. visible), and the size of phasmids (very small at the posterior part of the tail vs. inconspicuous). When compared to *H. egyptii*, *H. casmirica* n. sp. differs in IJ tail length (85–115 vs. 53–75 µm), anterior end to excretory pore distance (98–129 vs. 81–94 µm),* c* ratio (4.7–6.4 vs. 6.8–9.1), and *D*% (83–97 vs. 74–82). When compared to *H. georgiana*, *H. casmirica* n. sp. IJs exhibit distinctions in visibility of the bacterial cell (invisible vs. visible posterior to cardia), with that in J2 and J3 occupying more than one half of the tail length (vs. about one half), and in phasmid size (very small at the posterior part of the tail vs. inconspicuous). When compared to *H. ruandica*, *H. casmirica* n. sp. IJs can be distinguished by the anterior end to nerve ring distance (79–94 vs. 52–64 µm), the position of the excretory pore (at isthmus level vs. at or just posterior to the basal bulb), tail length (85–115 vs. 49–64 µm), neck length (114–138 vs. 75–102 µm),* c* ratio (4.7–6.4 vs. 3.4–5.8), and presence of a cephalic tooth (small vs. large). When compared to *H. zacatecana*, the IJs of *H. casmirica* n. sp. differ in maximum body diameter (17–24 vs. 23–27 µm), the position of the excretory pore (at isthmus level vs. at or just posterior to the basal bulb), the anterior end to nerve ring distance (79–94 vs. 69–72 µm), neck length (114–138 vs. 78–99 µm), tail length (85–115 vs. 52–63 µm),* c* ratio (4.7–6.4 vs. 8.2–10), and* c*′ ratio (5.1–8.0 vs. 4.3–6.7). A detailed comparison of the morphology of the IJs of *H. casmirica* n. sp. with those of other *Heterorhabditis* species is given in Table 2.

The males of *H. casmirica* n. sp. can be distinguished from those of *H. bacteriophora* based on the neck length (106–118 vs. 99–105 µm),* b* ratio (6.4–8.2 vs. 9.1),* c*′ ratio (1.1–1.6 vs. 1.8), *D*% (99–107 vs 117 µm), spicules with a rectangular manubrium with strongly refringent walls (vs rectangular with scarcely refringent walls), gubernaculum more than a half of the spicule length (vs. shorter) and GP1 at the level of the manubrium (vs. more anterior in the type population). In comparison to male *H. beicherriana*, differences include body size (0.6–0.9 vs. 0.9–1.2 mm), maximum body diameter (24–48 vs. 51–73 μm), the distance from the anterior end to the excretory pore (102–120 vs. 130–157 μm), the distance from the anterior end to the nerve ring (58–80 vs. 81–108 μm), the tail length (16–32 vs. 32–45 μm), *D*% (99–107 vs. 102–120 µm), GP1 at spicule level (vs. more anterior), the shape of the spicule manubrium (quadrangular vs. oblongate) and gubernaculum (more than half of the spicule length vs. similar length). Compared to males of *H. egyptii*, differences lie in the* c* ratio (24–35 vs. 19.5). When compared to males of *H. georgiana*, differences lie in the position of the excretory pore (at bulb or intestine level vs. posterior to the basal bulb only), spicules with rectangular manubrium with strongly refringent walls (vs rectangular with scarcely refringent walls) and gubernaculum (more than a half of the spicule length vs. a half of the spicule length). Compared to males of *H. ruandica*, differences include the shape of the spicule manubrium (well developed, quadrangular and with strongly refringent walls vs. poorly developed, triangular and not refringent), the shape of the gubernaculum manubrium (hook-like vs. straight) and gubernaculum (more than a half of the spicule length vs. a half). Compared to males of *H. zacatecana*, differences include the shape of the spicule manubrium (quadrangular with strongly refringent walls vs. rounded and not refringent), bursa with GP1-GP2 distance shorter (less than the corresponding body diameter vs. slightly longer), GP2–GP3 slightly separated (vs. very closed), spicule manubrium (with angular anterior end vs. with rounded anterior end), the shape of the gubernaculum manubrium (hook-like vs. slightly curved) and gubernaculum more than a half of the spicule length (vs. shorter). Lastly, differences from males of *H. hambletoni* include the distance from the anterior end to the nerve ring (58–80 vs. 80–90 μm). With respect to the males of all of the other species, *H. casmirica* n. sp. has a different spicule morphology (manubrium with thick and refringent walls and lacking a dorsal hump vs. thin walls and a small dorsal hump) and gubernaculum with a hook-like manubrium (vs. straight).

The hermaphroditic females of *H. casmirica* n. sp. can be distinguished from those of *H. bacteriophora* based on several characteristics, including the distance from the anterior end to the nerve ring (77–100 vs. 121–130 μm), and a larger* V*% (46–57 vs. 41–47). The hermaphroditic females of the new species can be differentiated from those of *H. beicherriana* by the distance from the anterior end to the nerve ring (77–100 vs. 135–243 μm), and a smaller anal body diameter (36–56 vs. 51–92 µm). Additionally, hermaphroditic females of *H. casmirica* n. sp. differ from those of *H. egyptii* by the distance from the anterior end to the nerve ring (77–100 vs. 101–147 μm); from those of *H. georgiana* by the distance from the anterior end to the excretory pore (180–211 vs. 200–277 μm) and the distance from the anterior end to the nerve ring (77–100 vs. 143–217 μm); from those of *H. ruandica* in tail shape (conoid vs. conoid-elongate) and size (longer vs. short), visible uteri (vs. not well visible), the* a* ratio (19–27 vs. 12–16), and* c* ratio (56–84 vs. 34–51); from those of *H. zacatecana* by shorter length (0.28–0.42 vs. 0.44–0.62 mm), the distance from the anterior end to the nerve ring (77–100 vs. 96–169 μm), visible oviducts and uteri (vs. not well visible), and shorter neck length (154–176 vs. 174–231 µm).

Amphimictic females of *H. casmirica* n. sp. can be differentiated from those of *H. bacteriophora* by their shorter rectum (slightly longer than the anal body diameter vs. about three times longer in the type population), smaller phasmids (vs. inconspicuous), shorter length (0.13–2.0 vs. 0.32–0.39 mm), smaller maximum body diameter (73–150 vs. 160–220 μm), the distance from the anterior end to the excretory pore (135–157 vs. 174–214 μm), and demanian ratios. Compared to *H. beicherriana*, amphimictic females of *H. casmirica* n. sp. have a shorter tail (conoid vs. conoid-elongate), with an acute tip (vs. finely rounded tip), differ in their phasmids (very small vs. inconspicuous), and have a smaller anal body diameter (22–30 vs. 35–81 μm). In comparison to *H. egyptii*, amphimictic females of *H. casmirica* n. sp. have a shorter tail (conoid vs. conoid-elongate), longer distance from the anterior end to the excretory pore (135–157 vs. 69–106 μm), and longer neck length (132–156 vs. 106–125 μm). Additionally, amphimictic females of *H. casmirica* n. sp. differ from those of *H. georgiana* by having smaller phasmids (vs. inconspicuous), and from those of *H. ruandica* by having a longer neck (132–156 vs. 107–132 μm), different* a* ratio (14–15 vs. 15–20), and smaller phasmids (vs. inconspicuous). Finally, compared to amphimictic females of *H. zacatecana*, those of the new species have a smaller maximum body diameter (73–150 vs. 160–228 μm), different* b* ratio (10–13 vs. 16–21),* c* ratio (16–31 vs. 31–63), smaller phasmids (vs. inconspicuous), and smaller anal body diameter (22–30 vs. 31–41 μm). Summaries of the similarities and differences between males, hermaphroditic females, and amphimictic females of *H. casmirica* n. sp. and other *Heterorhabditis* species are presented in Tables 3, 4, 5, respectively.

### Life cycle

*Heterorhabditis casmirica* n. sp. is a highly pathogenic nematode species that can be easily raised on *G. mellonella* larvae at a temperature ranging from 18 to 24 °C. The life cycle of this new species is comparable to that of other *Heterorhabditis* species. When *G. mellonella* larvae are exposed to 50–100 IJs, they die within 36–48 h and appear bright reddish after 48–72 h. First- and second-generation adults of *H. casmirica* n. sp. can be found in the insect cadavers 5–6 and 7–9 days after infection, respectively. The pre-infective juveniles left the host body, matured for a few days, and then migrated to the water traps after 15–21 days.

### Type host and locality

The specific host for *H. casmirica* n. sp. is currently unknown as these nematodes were isolated from soil samples using the insect baiting technique [24, 77, 78]. *Heterorhabditis casmirica* n. sp. populations were collected from soil samples in the union territory of Jammu and Kashmir, located in the northwest region of India, and specifically in the Himalayan Pir Panjal region.

### Type material

The type material for *H. casmirica* n. sp. (holotype male, 15 hermaphroditic female paratypes, 15 male paratypes, 15 amphimictic female paratypes and 19 J3, all belonging to the HM population) were deposited in the National Nematode Collection of India, Indian Agricultural Research Institute, New Delhi. Nematode cultures are maintained at the Sher-e-Kashmir University of Agricultural Sciences and Technology of Kashmir, India.

### Etymology

The specific name “*casmirica*” is derived from the Kashmir division (Casmiria in Latin), the geographical region where the nematodes used to describe the new species were collected.

### Cross-hybridization experiments

No viable offspring were observed when *H. casmirica* n. sp. nematodes of the HM strain were allowed to interact with Indian populations of *H. bacteriophora*, *H. indica*, and *H. baujardi*. However, fertile progenies were observed when six different populations of *H.* *casmirica* n. sp. nematodes were allowed to interact, indicating that these populations are conspecific but reproductively isolated from closely related species, including *H. bacteriophora*, *H. indica*, and *H. baujardi*. Fertile progeny was also observed when all the nematode strains self-fertilized.

### Nematode molecular characterization

The six populations of *H. casmirica* n. sp. were molecularly characterized based on the sequences of various genetic regions, including the ITS region of the rRNA (NCBI accession numbers OQ517936–OQ517941), the D2–D3 expansion segments of 28S rRNA (NCBI accession numbers OQ517947–OQ517952), mitochondrial 12S rRNA (NCBI accession numbers OQ517975-OQ517980), and *MT-COI* (NCBI accession numbers OQ517969–OQ517974). The ITS region of *H. casmirica* n. sp. is 771 bp in length, with ITS1 comprising 389 bp, 5.8S comprising 154 bp, and ITS2 comprising 228 bp. The *MT-COI* region flanked by primers HCF and HCR of *H. casmirica* n. sp. shows sequence similarity scores ranging from 75 to 94% with other *Heterorhabditis* species, and differs in 17–57 nucleotide positions (Table 6). Considering this genetic region, *H. casmirica* n. sp. is closely related to *H. bacteriophora, H. ruandica,* and *H. zacatecana* (Table 6). *Heterorhabditis bacteriophora* and *H. ruandica* both share 94% similarity with *H. casmirica* n. sp. and differ in 17 nucleotide positions. *Heterorhabditis zacatecana* shares 93% similarity with *H. casmirica* n. sp., and differs in 21 nucleotide positions. Fewer differences between *H. casmirica* n. sp. and its more closely related species were observed in the rRNA gene sequences. When compared with *H. casmirica* n. sp., *H. bacteriophora* and *H. zacatecana* both share 99.7% similarity and differ in two nucleotide positions, while *H. ruandica* shares 99.5% similarity and differs in four nucleotide positions in the ITS rRNA sequences flanked by primers TW81 and AB28 (Additional file 1: Table S2). All these three species share 100% similarity in the D2–D3 rRNA sequences flanked by primers D2A and D3B (Additional file 1: Table S3). Currently, very few mitochondrial 12S rRNA gene sequences are publicly available for molecular comparisons and phylogenetic analysis. Nevertheless, the sequences obtained in this study were deposited in the NCBI database for future taxonomic studies.

**Table 6:** Pairwise distances (in base pairs) of the mitochondrially encoded cytochrome C oxidase subunit I gene (*MT-COI*) regions between *Heterorhabditis casmirica* n. sp and other species of *Heterorhabditis*

Species (*MT-COI*)	*Heterorhabditis casmirica* n. sp. (HM population)	*Heterorhabditis bacteriophora* TT01	*Heterorhabditis ruandica* Rw14_N-C4a	*Heterorhabditis zacatecana* MEX-39	*Heterorhabditis beicherriana* CD2516	*Heterorhabditis georgiana* CD2500	*Heterorhabditis zealandica* CD2507	*Heterorhabditis megidis* CD2518	*Heterorhabditis atacamensis* MEX-20	*Heterorhabditis marelatus*	*Heterorhabditis downesi* CD2508	*Heterorhabditis mexicana*	*Heterorhabditis floridensis* CD2503	*Heterorhabditis taysearae*	*Heterorhabditis amazonensis* CD2510	*Heterorhabditis baujardi* CD2519	*Heterorhabditis noenieputensis* CD2506	*Heterorhabditis indica* LN2
*Heterorhabditis casmirica* n. sp. (HM population)		*17*	*18*	*21*	*23*	*27*	*30*	*35*	*42*	*42*	*42*	*44*	*45*	*45*	*46*	*47*	*52*	*57*
*Heterorhabditis bacteriophora* TT01	*94*		19	19	21	23	31	32	42	40	35	36	41	37	41	39	44	50
*Heterorhabditis ruandica* Rw14_N-C4a	*94*	94		8	16	24	32	29	40	36	31	45	48	45	49	45	51	55
*Heterorhabditis zacatecana* MEX-39	*93*	94	98		21	24	31	31	41	37	32	44	47	46	48	46	51	54
*Heterorhabditis beicherriana* CD2516	*92*	93	95	93		22	26	27	36	30	31	44	44	42	47	41	48	49
*Heterorhabditis georgiana* CD2500	*91*	92	92	92	93		29	23	33	32	26	40	43	42	38	37	50	47
*Heterorhabditis zealandica* CD2507	*89*	89	89	89	91	90		24	34	30	34	39	40	42	41	35	46	46
*Heterorhabditis megidis* CD2518	*87*	89	90	89	91	92	92		33	28	21	39	41	42	42	35	52	41
*Heterorhabditis atacamensis* MEX-20	*84*	84	85	85	87	88	88	88		26	37	47	49	47	43	38	46	47
*Heterorhabditis marelatus*	*84*	85	87	86	89	89	89	90	91		34	45	48	48	47	42	50	48
*Heterorhabditis downesi* CD2508	*84*	87	89	89	89	91	88	93	86	88		41	41	42	42	35	53	47
*Heterorhabditis mexicana*	*83*	87	82	83	82	84	85	85	82	82	84		11	13	26	19	45	45
*Heterorhabditis floridensis* CD2503	*82*	84	81	82	82	83	85	84	81	81	84	97		18	33	26	47	43
*Heterorhabditis taysearae*	*82*	86	82	82	83	83	84	84	82	81	83	96	94		31	20	44	50
*Heterorhabditis amazonensis* CD2510	*82*	84	80	81	81	85	84	84	84	81	83	91	88	89		27	48	49
*Heterorhabditis baujardi* CD2519	*81*	85	83	82	84	86	87	87	86	84	87	94	91	94	91		45	40
*Heterorhabditis noenieputensis* CD2506	*77*	82	78	78	80	79	80	77	81	79	76	82	81	82	80	82		37
*Heterorhabditis indica* LN2	*75*	79	76	77	79	81	81	84	81	80	81	82	83	80	80	85	86	

Data for *H. casmirica* n. sp. are in italic. . Data below the diagonal indicate percentage similarity. Data above the diagonal indicate the total difference between the characters

### Nematode phylogenetic reconstructions

**Fig. 7:**
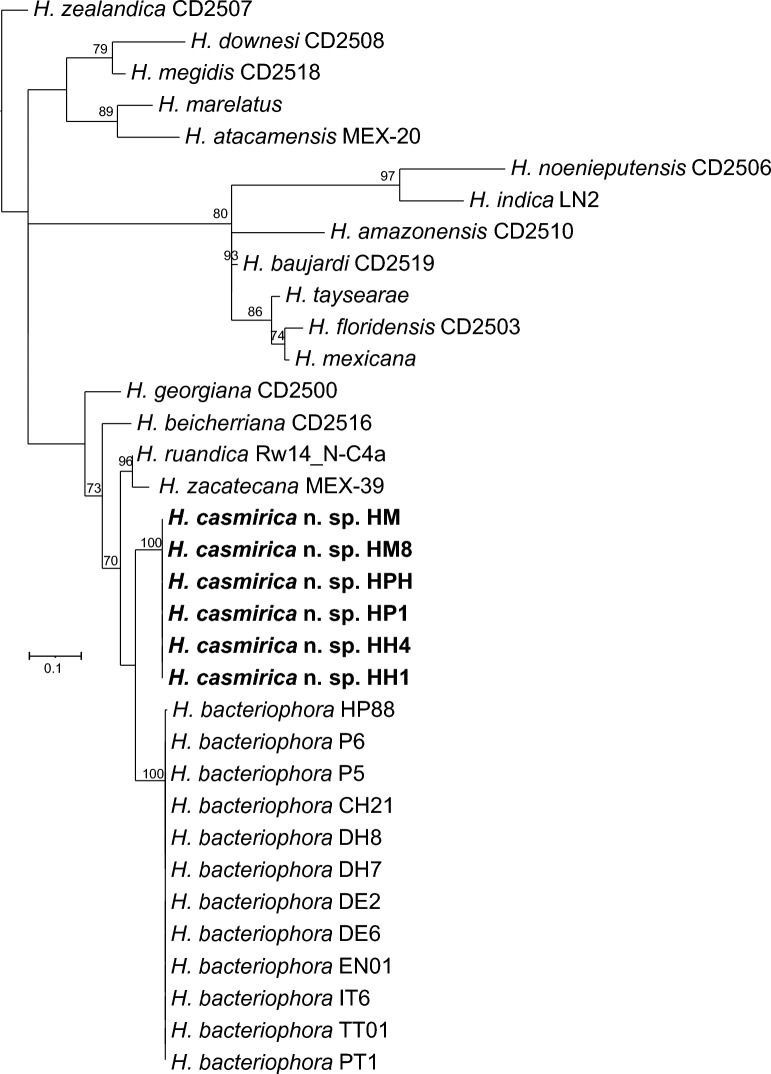
Maximum-likelihood phylogenetic tree between the newly described *Heterorhabditis casmirica* n. sp. and described species of *Heterorhabditis* based on nucleotide sequences of *MT-COI* flanked by primers HCF and HCR. Numbers at nodes represent bootstrap values based on 100 replications. Bars represent average nucleotide substitutions per sequence position. National Center for Biotechnology Information (NCBI) accession numbers of the nucleotide sequences used for the analyses are shown in Additional file 1: Table S4. The scale bar shows the number of substitutions per site

**Fig. 8:**
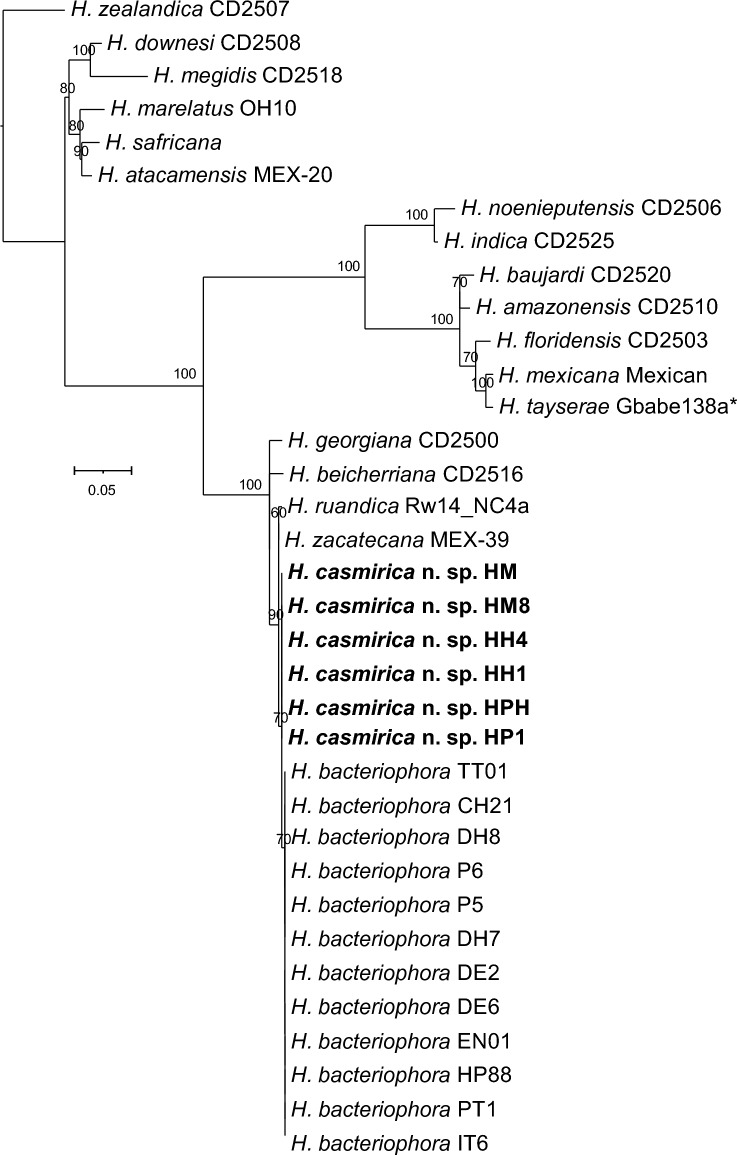
Maximum-likelihood phylogenetic tree between the newly described *Heterorhabditis casmirica* n. sp. and described species of *Heterorhabditis* based on nucleotide sequences of the internal transcribed spacer (ITS) (ITS1-5.8S-ITS2) ribosomal RNA (rRNA), flanked by primers 18S and 26S. Numbers at nodes represent bootstrap values based on 100 replications. Bars represent average nucleotide substitutions per sequence position. NCBI accession numbers of the nucleotide sequences used for the analyses are shown in Additional file 1: Table S4. The scale bar shows the number of substitutions per site

**Fig. 9:**
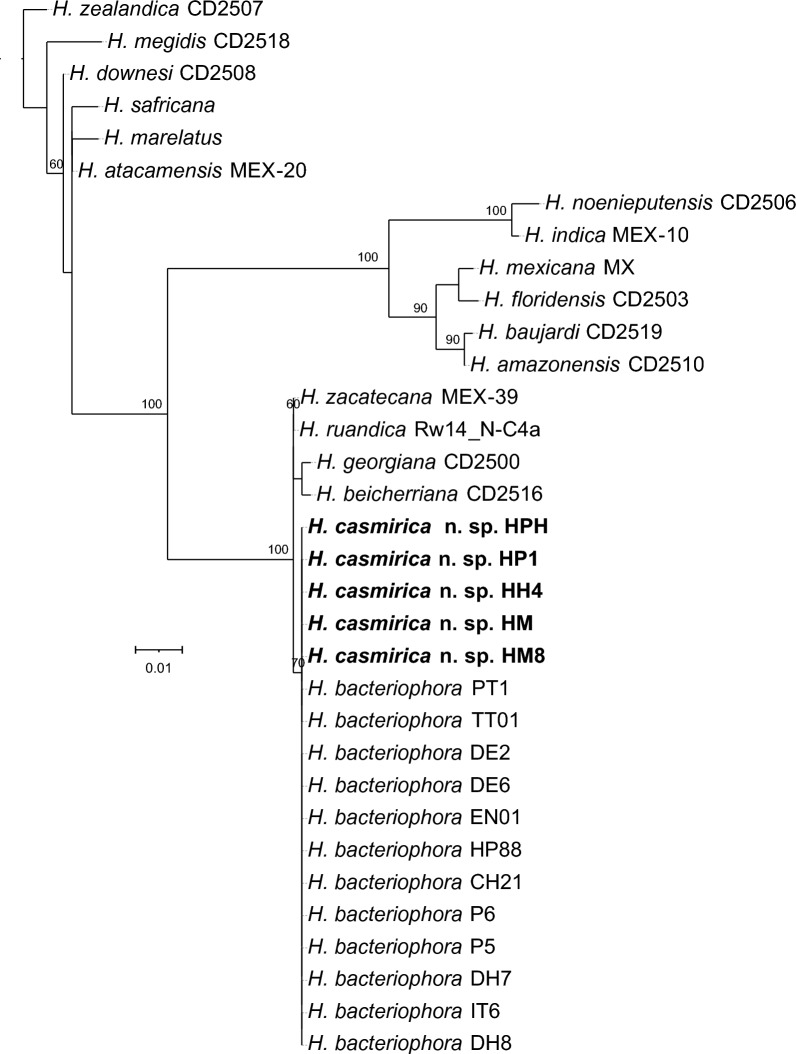
Maximum-likelihood phylogenetic tree reconstructed from the nucleotide sequences of the D2–D3 expansion segments of the 28S rRNA (D2–D3), flanked by primers D2A and D2B. Accession numbers of the nucleotide sequences used for the analyses are shown in Additional file 1: Table S4. Numbers at nodes represent bootstrap values based on 100 replications. Bars represent average nucleotide substitutions per sequence position

Phylogenetic analyses based on different genetic markers show that *H. casmirica* n. sp. belongs to the “bacteriophora” clade, which is currently composed of *H. bacteriophora*, *H. beicherriana, H. georgiana, H. ruandica,* and *H. zacatecana* (Figs. 7, 8, 9). *MT-COI* is particularly useful for the differentiation of all of these closely related species, and clearly shows that *H. casmirica* n. sp. and *H. bacteriophora*, its more closely related species, form two independent subclusters (Fig. 7). However, sequences of the ITS and D2–D3 regions of the rRNA gene, although allowing for the differentiation of certain species (Figs. 7, 8), provide lower phylogenetic resolving power than the *MT-COI* gene, as reported by Dhakal et al. [46] and Machado et al. [17]. Hence, *MT-COI* is particularly useful for the molecular discrimination of closely related species of the genus *Heterorhabditis*.

### Morphological and molecular relationships between *H. casmirica *n. sp. and specimens of *H. bacteriophora* present in India

At the morphological level, *H. casmirica* n. sp. differs from previously reported Indian isolates of *H. bacteriophora* [22, 30] (Additional file 1: Table S1). In particular, we observed that the males differ in spicule manubrium with strongly refringent walls (vs with scarcely refringent walls), gubernaculum more than a half of the spicule length (vs. shorter) and GP1 at manubrium level (vs. more anterior in the type population). The amphimictic females differ in smaller phasmids (vs. inconspicuous). The IJs differ in the distance from the anterior end to the nerve ring (79–94 vs. 48–74 µm), presence of bacterial sac (invisible vs. visible in the ventricular portion of the intestine), and size of phasmids (very small at posterior part of tail vs. inconspicuous) (Additional file 1: Table S1).

At the molecular level, *H. casmirica* n. sp. differs in 17 nucleotide positions in the* MT-COI* gene from several *H. bacteriophora* isolates from India, such as DH7, DH8, CH21, P5 and P6. On average, *H. casmirica* n. sp. shares 94% similarity with these isolates. In addition, the Indian populations of *H. bacteriophora* share 99.7% similarity with *H. casmirica* n. sp., and differ in two nucleotide positions in the ITS rRNA gene. Lastly, these two species do not differ in the sequences of the D2–D3 rRNA gene. Notably, the Indian populations DH7, DH8, CH21, P5 and P6 share 100% similarity with the type population of *H. bacteriophora* across all the gene markers used, and hence corroborate the conclusions of previous studies [22, 30]. The phylogenetic study further confirms the distinctiveness of the Indian populations of *H. bacteriophora* from *H. casmirica* n. sp. and establishes their similitude with the type population of *H. bacteriophora* (Figs. 7, 8, 9).

### Symbiotic relationships

**Fig. 10:**
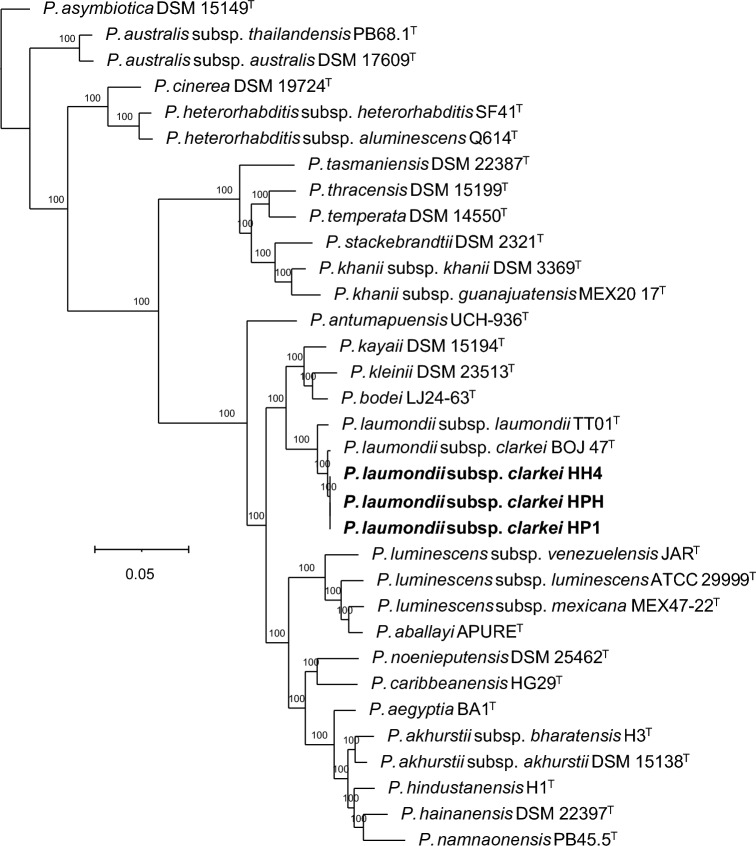
Phylogenetic reconstruction based on core genome sequences of *Photorhabdus* bacterial strains; 2,227,040 nucleotide positions (2216 core genes) were used in the analysis. Numbers at the nodes represent Shimodaira–Hasegawa-like branch supports. Bar represents average nucleotide substitutions per sequence position. NCBI accession numbers of the genome sequences used for the reconstruction are shown in Additional file 1: Table S5. The scale bar shows the number of substitutions per site

Phylogenetic reconstructions based on core genome sequences and sequence comparisons show that the bacterial symbionts isolated from *H. casmirica* n. sp. are very similar and belong to the subspecies *Photorhabdus laumondii* subsp. *clarkei* (Fig. 10). The digital DNA–DNA hybridization (dDDH) scores between BOJ47^T^, the type strain of the species *P. laumondii* subsp. *clarkei*, and strains HH4, HPH, and HP1, isolated from *H. casmirica* n. sp. HH4, HPH and HP1, are 94.3%, which is above the 70 and 79% thresholds that delimit prokaryotic species and subspecies, and confirms that they are conspecific [79].

### A side note on the nomenclature of *Heterorhabditis marelatus*

The term “*marelatus*” was created by combining the Latin words “*mare*” meaning sea and “*latus*” meaning side in an attempt to translate the type locality “seaside” into Latin [70]. Hence, *marelatus* was formed as a noun, not as an adjective. Sudhaus [80] changed the specific epithet of the species *Heterorhabditis marelatus* to “*marelata.*” This change was perhaps motivated by the fact that the genus noun *Heterorhabditis* is feminine and that, in Latin, the specific epithet should agree in gender with the genus. However, nouns in Latin do not vary according to gender, and therefore we propose that the correct term is “*marelatus*.” Hence, we propose that the original species nomenclature, *Heterorhabditis marelatus*, should be maintained.

## Conclusions

Six populations of *Heterorhabditis* nematodes were identified in the present study that exhibited clear distinctions in their morphology, morphometric and molecular characteristics, as well as reproductive isolation and phylogenetic separation from all known *Heterorhabditis* species. We propose the name* Heterorhabditis casmirica* n. sp. for this new species, which is the second new *Heterorhabditis* entomopathogenic nematode species reported from the Indian subcontinent. Our results highlight the importance of using both classical taxonomy and molecular markers (*MT-COI*, ITS, small subunit, and large subunit) to accurately describe new *Heterorhabditis* species and their bacterial symbionts. The discovery of *H. casmirica* n. sp. and its associated bacterial symbiont expands our understanding of the biodiversity and distribution of these biocontrol agents and underscores their potential in the development of new biocontrol strategies against insect pests.

### Supplementary Information

**Additional file 1: Table S1.** Comparative morphometrics of infective juveniles and adult generations of *Heterorhabditis casmirica* n. sp. with type populations of *Heterorhabditis bacteriophora* and Indian strains. All data, with the exception of ratios and percentages, are given in micrometers and as mean (range). **Table S2.** Pairwise distances in base pairs of the ITS rRNA regions between species of *Heterorhabditis* and *Heterorhabditis casmirica* n. sp. Data for *H. casmirica* n. sp. are in italic. **Table S3.** Pairwise distances in base pairs of the D2–D3 rRNA regions between species of *Heterorhabditis* and *Heterorhabditis casmirica* n. sp. Data for *H. casmirica *n. sp. are in italic. **Table S4.** National Center for Biotechnology Information (NCBI) accession numbers of the nucleotide sequences used for the phylogenetic analyses in this study; the sequences newly generated in this study are in italic. **Table S5**. NCBI accession numbers of the genomic sequences of different *Photorhabdus* species used in this study; the sequences newly generated in this study are in italic.

## Data Availability

Our sequences were deposited in the GenBank database under the accession numbers given in Additional file 1: Tables S3 and S4. Data supporting the conclusions of this article are included within the article. The datasets used and/or analyzed during the current study are available from the corresponding author upon reasonable request.

## Abbreviations

GP: Genital papilla
IJs: Infective juveniles
ITS: Internal transcribed spacer
LB: Luria—Bertani
LM: Light microscopy
*MT-COI*: Mitochondrially encoded cytochrome C oxidase subunit I
NCBI: National Center for Biotechnology Information
SEM: Scanning electron microscopy
