# Correction: Taxonomic and molecular characterization of a new entomopathogenic nematode species, *Heterorhabditis casmirica* n. sp., and whole genome sequencing of its associated bacterial symbiont

**DOI:** 10.1186/s13071-023-06060-0

**Published:** 2023-11-27

**Authors:** Aashaq Hussain Bhat, Ricardo A. R. Machado, Joaquín Abolafia, Alba N. Ruiz-Cuenca, Tarique Hassan Askary, Fuad Ameen, Wasim Muzamil Dass

**Affiliations:** 1 Department of Biosciences, University Center for Research and Development, Chandigarh University, Gharuan, Mohali, Punjab 140413 India; 2 Experimental Biology Research Group, Institute of Biology, Faculty of Sciences, University of Neuchâtel, 2000 Neuchâtel, Switzerland; 3 Departamento de Biología Animal, Biología Vegetal y Ecología, Universidad de Jaén, Campus ‘Las Lagunillas’, 23071 Jaén, Spain; 4 Division of Entomology, Faculty of Agriculture, Sher-e-Kashmir University of Agricultural Sciences and Technology of Kashmir, Wadura Campus, Sopore, 193201 Jammu and Kashmir India; 5 Department of Botany and Microbiology, College of Science, King Saud University, 11451 Riyadh, Saudi Arabia; 6 Department of Zoology, University of Kashmir, Srinagar, 190006 Jammu and Kashmir India

**Correction** **:** **Parasites & Vectors (2023) 16:383** **https://doi.org/10.1186/s13071-023-05990-z**

Following publication of the original article [1], it came to the attention of the authors that the scientific name of the symbiont associated with *Heterorhabditis casmirica* nematodes was incorrectly written: while it should be *Photorhabdus laumondii* subsp. *clarkei*, as it is on the phylogenetic tree (Fig. 10), *Photorhabdus luminescence* subsp. *clarkei* was instead written, namely in the Abstract (Page 1) and in the ‘Symbiotic relationships’ subsection to be found on pages 24 and 25 of the article PDF. The name has since been corrected in the published article. The authors thank you for reading this erratum and apologize for any inconvenience caused.
