# Supplementary material for: Taxonomic and molecular characterization of a new entomopathogenic nematode species, Heterorhabditis casmirica n. sp., and whole genome sequencing of its associated bacterial symbiont
Source: Parasit Vectors. 2023 Oct 25;16:383. doi: 10.1186/s13071-023-05990-z (PMC10598981; doi:10.1186/s13071-023-05990-z)
Supplement: Supplementary file 1 — Additional file 1: Table S1. Comparative morphometrics of infective juveniles and adult generations of Heterorhabditis casmirica n. sp. with type populations of Heterorhabditis bacteriophora and Indian strains. All data, with the exception of ratios and percentages, are given in micrometers and as mean (range). Table S2. Pairwise distances in base pairs of the ITS rRNA regions between species of Heterorhabditis and Heterorhabditis casmirica n. sp. Data for H. casmirica n. sp. are in italic. Table S3. Pairwise distances in base pairs of the D2–D3 rRNA regions between species of Heterorhabditis and Heterorhabditis casmirica n. sp. Data for H. casmirica n. sp. are in italic. Table S4. National Center for Biotechnology Information (NCBI) accession numbers of the nucleotide sequences used for the phylogenetic analyses in this study; the sequences newly generated in this study are in italic. Table S5. NCBI accession numbers of the genomic sequences of different Photorhabdus species used in this study; the sequences newly generated in this study are in italic. [file 13071_2023_5990_MOESM1_ESM.docx]

**TABLE S1.** Comparative morphometrics of infective juveniles and adult generations of *Heterorhabditis casmirica* n. sp. with type populations of *Heterorhabditis bacteriophora* and Indian strains. All measurements are in µm (except ratio and percentage) and in the form of mean (range).

| **Infective juveniles** | **L** | **BD** | **EP** | **NR** | **PS** | **T** | ***a*** | ***b*** | ***c*** | **D%** | **E%** |
| --- | --- | --- | --- | --- | --- | --- | --- | --- | --- | --- | --- |
| *H. bacteriophora* DH7 | 453–617 | 19–27 | 72–102 | 50–74 | 83–106 | 47–89 | 19–29 | 4.9–7.4 | 6.0–12 | 78–107 | 105–189 |
| *H. bacteriophora* DH8 | 206–832 | 18–26 | 70–101 | 47–70 | 78–111 | 50–75 | 17–35 | 2.0–8.6 | 6.2–13 | 83–105 | 113–193 |
| *H. bacteriophora* P5 | 471–571 | 22–28 | 65–102 | 48–69 | 79–110 | 57–79 | 19–25 | 4.7–6.1 | 5.5–9.3 | 81–109 | 111–181 |
| *H. bacteriophora* | 512–671 | 18–31 | 87–110 | 72–93 | 101–139 | 83–112 | 17–30 | 4.0–5.1 | 5.7–7.0 | 76–92 | 103–130 |
| ***H. casmirica* n. sp.** | **512–599** | **17–24** | **98–129** | **79–94** | **99–127** | **85–115** | **20–25** | **4.0–5.2** | **5.1–8.0** | **83–97** | **93–136** |
| **Males** | **L** | **BD** | **EP** | **NR** | **PS** | **T** | **SL** | **GL** | **SW%** | **GS%** | **D%** |
| *H. bacteriophora* DH7 | 805–1075 | 42–57 | 84–111 | 48–75 | 80–119 | 24–39 | 39-51 | 17–27 | 170–225 | 40–62 | 77–136 |
| *H. bacteriophora* DH8 | 735–892 | 36–50 | 78–95 | 59–78 | 96–109 | 21–29 | 36-51 | 18–25 | 168–309 | 38–62 | 78–94 |
| *H. bacteriophora* P5 | 762–930 | 49–64 | 83–103 | 57–72 | 81–105 | 25–31 | 31–50 | 17–26 | 174–252 | 39–54 | 78–96 |
| *H. bacteriophora* | 780–960 | 38–46 | 114–130 | 65–81 | 99–105 | 22–36 | 36-44 | 18–25 | 174 | 50 | 117 |
| ***H. casmirica* n. sp.** | **608–914** | **24–48** | **102–120** | **58–80** | **96–114** | **16–32** | **38–48** | **18–26** | **160–252** | **45–63** | **99–107** |
| **Hermaphrodites** | **L** | **BD** | **EP** | **NR** | **PS** | **T** | **V** | **D%** | **E%** | **ABW** | **WEP** |
| *H. bacteriophora* DH7 | 3916–5155 | 205–266 | 153–198 | 94–127 | 158–207 | 70–98 | 37–46 | 75–103 | 188–258 | 39–51 | 103–158 |
| *H. bacteriophora* DH8 | 3747–5753 | 236–334 | 140–288 | 87–143 | 154–204 | 80–128 | 41–54 | 78–168 | 115–268 | 53–75 | 105–158 |
| *H. bacteriophora* P5 | 3072–5382 | 221–332 | 123–251 | 80–128 | 103–208 | 69–105 | 39–54 | 79–146 | 145–292 | 44–71 | 74–124 |
| *H. bacteriophora* | 3630–4390 | 160–180 | 189–217 | 121–130 | 189–205 | 81–93 | 41–47 | 106 | 232 | 40–53 | – |
| ***H. casmirica* n. sp.** | **2851–4219** | **140–341** | **180–211** | **77–100** | **168–202** | **72–114** | **46–57** | **94–120** | **205–292** | **36–56** | **71–108** |
| **Females** | **L** | **BD** | **EP** | **NR** | **PS** | **T** | **V** | **D%** | **E%** | **ABW** | **WEP** |
| *H. bacteriophora* DH7 | 1226–1819 | 58–115 | 108–157 | 68–91 | 118–141 | 29–94 | 44–58 | 83–116 | 126–466 | 24–31 | 43–80 |
| *H. bacteriophora* DH8 | 1230–1822 | 55–88 | 106–158 | 64–89 | 101–127 | 30–93 | 43–53 | 82–110 | 127–324 | 20–51 | 42–59 |
| *H. bacteriophora* P5 | 1318–2890 | 54–115 | 108–141 | 66–93 | 102–128 | 32–89 | 46–54 | 88–115 | 127–330 | 22–34 | 47–82 |
| *H. bacteriophora* | 3180–3850 | 160–220 | 174–214 | 93–118 | 155–183 | 71–93 | 42–53 | 114 | 234 | 22–31 | – |
| ***H. casmirica* n. sp.** | **1273–1990** | **73–150** | **135–157** | **84–111** | **126–149** | **64–83** | **45–52** | **99–116** | **156–209** | **22–30** | **41–61** |

– = Character unknown.

**Table S2.** Pairwise distances in base pairs of the ITS rRNA regions among species of *Heterorhabditis* and *Heterorhabditis casmirica* n. sp. Data for new species is in bold.

| Species (ITS rRNA) | ***H. casmirica* n. sp. HM** | *H. bacteriophora* TT0101 | *H. zacatecana* MEX-3939 | *H. ruandica* Rw14_NC4a _NC4a | H._beicherriana_CD2516 | *H. georgiana* Hbb | *H. atacamensis* MEX-20 | *H. marelatus* OH10 | *H. safricana* | *H. downesi* CD2508 | *H. indica* CD2525 | *H. noenieputensis CD2506* | *H. megidis* CD2518 | *H. zealandica* CD2507 | *H. amazonensis* CD2510 | *H. baujardi* CD2519 | *H. mexicana* | *H. floridensis* CD250303 | *H. taysearae* Gbabe138a |
| --- | --- | --- | --- | --- | --- | --- | --- | --- | --- | --- | --- | --- | --- | --- | --- | --- | --- | --- | --- |
| ***H. casmirica* n. sp. HM** |  | **2** | **2** | **4** | **14** | **14** | **99** | **103** | **106** | **108** | **120** | **121** | **127** | **134** | **141** | **145** | **148** | **149** | **149** |
| *H. bacteriophora* TT01 | **99.7** |  | 4 | 6 | 16 | 16 | 97 | 101 | 104 | 106 | 122 | 123 | 125 | 133 | 140 | 143 | 147 | 147 | 148 |
| *H. zacatecana* MEX-39 | **99.7** | 99.5 |  | 2 | 14 | 12 | 101 | 103 | 107 | 109 | 119 | 120 | 126 | 136 | 140 | 144 | 147 | 148 | 148 |
| *H. ruandica* Rw14_N-C4a | **99.5** | 99.2 | 99.7 |  | 16 | 14 | 103 | 105 | 109 | 111 | 119 | 119 | 128 | 138 | 140 | 144 | 147 | 148 | 148 |
| *H. beicherriana* CD2516 | **98.1** | 97.8 | 98.1 | 97.8 |  | 16 | 103 | 105 | 109 | 111 | 125 | 126 | 130 | 137 | 146 | 150 | 153 | 154 | 154 |
| *H. georgiana* Hbb | **98.1** | 97.8 | 98.3 | 98.1 | 97.8 |  | 104 | 107 | 108 | 113 | 120 | 121 | 128 | 137 | 141 | 145 | 148 | 150 | 149 |
| *H. atacamensis* MEX-20 | **84.1** | 84.5 | 83.8 | 83.4 | 83.4 | 83.2 |  | 21 | 13 | 27 | 133 | 135 | 49 | 65 | 147 | 151 | 152 | 153 | 151 |
| *H. marelatus* OH10 | **83.4** | 83.7 | 83.3 | 83.0 | 83.0 | 82.6 | 97.0 |  | 24 | 35 | 140 | 142 | 55 | 68 | 151 | 155 | 157 | 158 | 158 |
| *H. safricana* | **82.8** | 83.2 | 82.6 | 82.3 | 82.2 | 82.4 | 98.1 | 96.5 |  | 30 | 140 | 141 | 51 | 72 | 155 | 158 | 160 | 161 | 159 |
| *H. downesi* CD2508 | **82.3** | 82.6 | 82.1 | 81.7 | 81.7 | 81.2 | 96.0 | 94.8 | 95.6 |  | 142 | 143 | 38 | 71 | 153 | 156 | 157 | 158 | 156 |
| *H. indica* CD2525 | **79.4** | 79.0 | 79.6 | 79.6 | 78.5 | 79.4 | 76.9 | 75.4 | 75.4 | 74.7 |  | 15 | 160 | 165 | 79 | 87 | 87 | 86 | 87 |
| *H. noenieputensis* CD2506 | **78.7** | 78.3 | 78.9 | 79.1 | 77.8 | 78.7 | 75.9 | 74.3 | 74.5 | 73.8 | 97.8 |  | 161 | 167 | 87 | 97 | 93 | 89 | 93 |
| *H. megidis* CD2518 | **78.7** | 79.1 | 78.9 | 78.5 | 78.1 | 78.4 | 92.7 | 91.7 | 92.3 | 94.4 | 71.1 | 70.1 |  | 88 | 170 | 171 | 174 | 174 | 173 |
| *H. zealandica* CD2507 | **77.5** | 77.7 | 77.1 | 76.7 | 76.9 | 76.8 | 90.0 | 89.5 | 88.8 | 88.9 | 70.0 | 68.6 | 86.0 |  | 178 | 180 | 182 | 182 | 184 |
| *H. amazonensis* CD2510 | **76.3** | 76.5 | 76.5 | 76.5 | 75.4 | 76.3 | 74.9 | 74.0 | 73.2 | 73.1 | 87.5 | 85.8 | 69.6 | 68.3 |  | 15 | 32 | 38 | 31 |
| *H. baujardi* CD2519 | **75.6** | 76.0 | 75.7 | 75.7 | 74.6 | 75.6 | 74.1 | 73.2 | 72.6 | 72.5 | 86.1 | 84.0 | 69.4 | 67.9 | 97.9 |  | 37 | 39 | 37 |
| *H. mexicana* Mexican | **74.6** | 74.8 | 74.7 | 74.7 | 73.6 | 74.6 | 73.6 | 72.5 | 71.9 | 72.0 | 86.0 | 84.6 | 68.4 | 67.1 | 95.4 | 94.7 |  | 20 | 9 |
| *H. floridensis* CD2503 | **74.4** | 74.8 | 74.5 | 74.5 | 73.4 | 74.2 | 73.4 | 72.3 | 71.7 | 71.8 | 86.1 | 85.3 | 68.4 | 67.1 | 94.5 | 94.3 | 97.2 |  | 20 |
| *H. taysearae* Gbabe138a | **74.3** | 74.6 | 74.5 | 74.5 | 73.4 | 74.4 | 73.8 | 72.2 | 72.0 | 72.2 | 85.9 | 84.6 | 68.6 | 66.6 | 95.5 | 94.6 | 98.8 | 97.2 |  |

Below diagonal: percentage similarity; above diagonal: total character difference.

**Table S3.** Pairwise distances in base pairs of the D2D3 rRNA regions among species of *Heterorhabditis* and *Heterorhabditis casmirica* n. sp. Data for new species is in bold.

| Species (D2D3 rRNA) | ***H. casmirica* n. sp. HM** | *H. bacteriophora* TT01 | *H. ruandica* Rw18_M-Hr1b | *H. zacatecana* MEX-39 | *H. georgiana* CD2500 | *H. beicherriana* 2516 | *H. atacamensis* MEX-20 | *H. downesi* CD2508 | *H. marelatus* | *H. safricana* | *H. zealandica* CD2507 | *H. mexicana* MX | *H. megidis* CD2518 | *H. floridensis* CD2503 | *H. amazonensis* CD2510 | *H. baujardi* CD2519 | *H. noenieputensis* CD2506 | *H. indica* MEX-10 |
| --- | --- | --- | --- | --- | --- | --- | --- | --- | --- | --- | --- | --- | --- | --- | --- | --- | --- | --- |
| ***H. casmirica* n. sp. HM** |  | **0** | **1** | **1** | **3** | **3** | **23** | **24** | **26** | **26** | **27** | **30** | **31** | **32** | **33** | **34** | **38** | **39** |
| *H. bacteriophora* TT01 | **100** |  | 1 | 1 | 3 | 3 | 23 | 24 | 26 | 26 | 27 | 30 | 31 | 32 | 33 | 34 | 38 | 39 |
| *H. ruandica* Rw14_NC4a | **99.8** | 99.8 |  | 0 | 2 | 2 | 22 | 23 | 25 | 25 | 26 | 31 | 30 | 33 | 34 | 35 | 37 | 38 |
| *H. zacatecana* MEX-39 | **99.8** | 99.8 | 100 |  | 2 | 2 | 22 | 23 | 25 | 25 | 26 | 31 | 30 | 33 | 34 | 35 | 37 | 38 |
| *H. georgiana* CD2500 | **99.4** | 99.4 | 99.6 | 99.6 |  | 2 | 24 | 25 | 27 | 27 | 28 | 33 | 32 | 35 | 36 | 37 | 39 | 40 |
| *H. beicherriana* CD2516 | **99.4** | 99.4 | 99.6 | 99.6 | 99.6 |  | 22 | 23 | 25 | 25 | 26 | 31 | 30 | 33 | 34 | 35 | 37 | 38 |
| *H. atacamensis* MEX-20 | **95.1** | 95.1 | 95.3 | 95.3 | 94.8 | 95.3 |  | 1 | 3 | 3 | 8 | 30 | 9 | 34 | 33 | 34 | 40 | 37 |
| *H. downesi* CD2508 | **94.8** | 94.8 | 95.1 | 95.1 | 94.6 | 95.1 | 99.8 |  | 4 | 4 | 7 | 29 | 8 | 33 | 32 | 33 | 41 | 38 |
| *H. marelatus* | **94.4** | 94.4 | 94.6 | 94.6 | 94.1 | 94.6 | 99.4 | 99.3 |  | 6 | 11 | 33 | 12 | 37 | 36 | 37 | 43 | 40 |
| *H. safricana* | **94.4** | 94.4 | 94.6 | 94.6 | 94.1 | 94.6 | 99.4 | 99.2 | 98.9 |  | 11 | 32 | 12 | 36 | 35 | 36 | 38 | 35 |
| *H. zealandica* CD2507 | **94.1** | 94.1 | 94.3 | 94.3 | 93.8 | 94.3 | 98.4 | 98.7 | 97.8 | 97.8 |  | 34 | 11 | 38 | 37 | 38 | 46 | 43 |
| *H. mexicana* MX | **93.0** | 93.0 | 92.8 | 92.8 | 92.2 | 92.8 | 93.2 | 93.5 | 92.4 | 92.7 | 92.0 |  | 37 | 4 | 7 | 7 | 21 | 19 |
| *H. megidis* CD2518 | **93.1** | 93.1 | 93.3 | 93.3 | 92.8 | 93.3 | 98.3 | 98.5 | 97.7 | 97.7 | 97.8 | 91.3 |  | 41 | 40 | 41 | 47 | 44 |
| *H. floridensis* CD2503 | **92.5** | 92.5 | 92.2 | 92.2 | 91.6 | 92.2 | 92.1 | 92.4 | 91.3 | 91.6 | 90.9 | 99.3 | 90.1 |  | 7 | 7 | 22 | 20 |
| *H. amazonensis* CD2510 | **92.2** | 92.2 | 91.9 | 91.9 | 91.3 | 91.9 | 92.4 | 92.7 | 91.6 | 91.8 | 91.2 | 98.7 | 90.4 | 98.7 |  | 1 | 23 | 21 |
| *H. baujardi* CD2519 | **91.8** | 91.8 | 91.6 | 91.6 | 91.0 | 91.6 | 92.1 | 92.4 | 91.3 | 91.5 | 90.8 | 98.7 | 90.1 | 98.7 | 99.8 |  | 23 | 21 |
| *H. noenieputensis* CD2506 | **90.9** | 90.9 | 91.2 | 91.2 | 90.6 | 91.2 | 90.3 | 90.0 | 89.5 | 90.9 | 88.3 | 95.6 | 88.2 | 95.4 | 95.2 | 95.1 |  | 4 |
| *H. indica* MEX-10 | **90.6** | 90.6 | 90.9 | 90.9 | 90.3 | 90.9 | 91.4 | 91.1 | 90.5 | 91.9 | 89.5 | 96.1 | 89.3 | 95.9 | 95.6 | 95.6 | 99.3 |  |

Below diagonal: percentage similarity; above diagonal: total character difference.

**Table S4**. National Center for Biotechnology Information (NCBI) accession numbers of the nematode sequences used in this study. In bold font are the sequences newly generated in this study.

| **S. No.** | **Organism** | | **Genetic Region** | | |
| --- | --- | --- | --- | --- | --- |
|  | **Species** | **Strain designation (s)** | **COI** | **ITS** | **D2D3** |
| 1. | *H. amazonensis* | CD2510, MC01 | MT372499 | MT372499 | MT372502 |
| 2. | *H. atacamensis* | MEX-20 | MW817979 | MK421485 | MW817561 |
| 3. | *H. bacteriophora* | TT01 | MW817975 | MZ326041 | MW817557 |
|  |  | DE2 | MW817970 | MZ326036 | MW817552 |
|  |  | DE6 | MW817971 | MZ326037 | MW817553 |
|  |  | EN01 | MW817972 | MZ326038 | MW817554 |
|  |  | CD2504, HP88 | MT373729 | MT372491 | MT372509 |
|  |  | PT1 | MW817974 | MZ326040 | MW817556 |
|  |  | IT6 | MW817973 | MZ326039 | MW817555 |
|  |  | DH7 | **OR415599** | MG559563 | **MK629947** |
|  |  | DH8 | **OR415600** | MK629980 | **MH298619** |
|  |  | CH21 | **OR415601** | MK072810 | **MK064159** |
|  |  | P5 | **OR415602** | MK256378 | **OR398615** |
|  |  | P6 | **OR415603** | MK263023 | **OR398616** |
| 4. | *H. baujardi* | CD2519, F10 | MT372500 | MT373736 | MT372503 |
| 5. | *H. beicherriana* | CD2516, QZL-2011 | MT372490 | MT373730 | MT372511 |
| **6.** | ***H. casmirica* n. sp.** | **HM, HM8, HP1, HPH, HH1, and HH4** | **OQ517936-OQ517941** | **OQ517969-OQ517974** | **OQ517947-OQ517952** |
| 7. | *H. downesi* | CD2508, 23.9 | MT372494 | MT373732 | MT372507 |
| 8. | *H. floridensis* | CD2503, K22 | MT372501 | MT373737 | MT372504 |
| 9. | *H. georgiana* | CD2500 | MT372492 | MT373731 | MT372510 |
|  | *H. indica* | CD2525 | MT372498 | - | - |
| 10. |  | LN2 | - | AB355853 | - |
|  |  | MEX-10 | - | - | MK421439 |
|  | *H. marelatus* | OH10 | AY321479 | - | - |
| 11. |  | No information | - | EF043419 | - |
|  |  | No information | - | - | EU100412 |
| 12. | *H. megidis* | CD2518, DV | MT372495 | MT373733 | MT372506 |
|  | *H. mexicana* | Mexican | AY321478 | - | - |
| 13. |  | No information | - | EF043422 | - |
|  |  | MX | - | - | EU100414 |
| 14. | *H. noenieputensis* | CD2506, SF669 | MT372497 | MT373728 | MT372505 |
| 15 | *H. ruandica* | Rw14_N-C4a | MZ326035 | MW817969 | MW817551 |
| 16. | *H. safricana* | No information | EF488006 | - | EU100416 |
| 17. | *H. taysearae* | Gbabe138a | MF372596 | - | - |
|  |  | No information | - | EF043421 | - |
| 18. | *H. zacatecana* | MEX-39 | MZ326030 | MW817964 | MW817546 |
| 19. | *H. zealandica* | CD2507 | MT372493 | MT373734 | MT372508 |

**– =** Accession numbers unknown.

**Table S5**. National Center for Biotechnology Information (NCBI) accession numbers of the bacterial sequences used in this study. In bold font are the sequences newly generated in this study.

| **S. No.** | **Strain** | **Genome** |
| --- | --- | --- |
| 1. | *P. aballayi* APURE^T^ | JAPFCD01 |
| 2. | *P. aegyptia* BA1^T^ | JFGV01 |
| 3. | *P. akhurstii* subsp. *akhurstii* DSM 15138^T^ | RCWE01 |
| 4. | *P. akhurstii* subsp*. bharatensis* H3^T^ | PUWU01 |
| 5. | *P. antumapuensis* sp. nov. UCH-936^T^ | JAHZMK01 |
| 6 | *P. australis* subsp*. thailandensis* PB68.1^T^ | LOMY01 |
| 7. | *P. australis* subsp*. australis* DSM 17609^T^ | JONO01 |
| 8. | *P. asymbiotica* ATCC 43949^T^ | RBLJ01 |
| 9. | *P. bodei* LJ24-63^T^ | NSCM01 |
| 10. | *P. caribbeanensis* DSM 22391^T^ | RCWB01 |
| 11. | *P. cinerea* DSM 19724^T^ | PUJW01 |
| 12. | *P. hainanensis* DSM 22397^T^ | RCWD01 |
| 13. | *P. heterorhabditis* subsp*. aluminescens* Q614^T^ | JABBCS01 |
| 14. | *P. heterorhabditis* subsp*. heterorhabditis* SF41^T^ | RCWA01 |
| 15. | *P. hindustanensis* H1^T^ | PUWT01 |
| 16. | *P. kayaii* DSM 15194^T^ | JAJAFZ01 |
| 17. | *P. khanii* subsp. *khanii* DSM 3369^T^ | AYSJ01 |
| 18. | *P. khanii* subsp. g*uanajuatensis* MEX20-17^T^ | PUJY01 |
| 19. | *P. kleinii* DSM 23513^T^ | JAJAFY01 |
| 20. | *P. laumondii* subsp. *clarkei* BOJ-47^T^ | NSCI01 |
| **21.** | ***P. laumondii* subsp. *clarkei* HH4** | **JARYLG01** |
| **22.** | ***P. laumondii* subsp. *clarkei* HP1** | **JARYLE01** |
| **23.** | ***P. laumondii* subsp. *clarkei* HPH** | **JARYLF01** |
| 24. | *P. laumondii* subsp. *laumondii* TT01^T^ | WSFH01 |
| 25. | *P. luminescens* subsp. *luminescens* ATCC 29999^T^ | FMWJ01 |
| 26. | *P. luminescens* subsp. *mexicana* MEX47-22^T^ | PUJX01 |
| 27. | *P. luminescens* subsp. *venezuelensis* JAR^T^ | JAPFFZ01 |
| 28. | *P. namnaonensis* PB45.5^T^ | LOIC01 |
| 29. | *P. noenieputensis* DSM 25462^T^ | RCWC01 |
| 30. | *P. stackebrandtii* DSM 23271^T^ | PUJV01 |
| 31. | *P. tasmaniensis* DSM 22387^T^ | PUJU01 |
| 32. | *P. temperata* DSM 14550^T^ | JAJAFX01 |
| 33. | *P. thracensis* DSM 15199^T^ | CP011104 |
